# Potential COVID-19 Therapies from Computational Repurposing of Drugs and Natural Products against the SARS-CoV-2 Helicase

**DOI:** 10.3390/ijms23147704

**Published:** 2022-07-12

**Authors:** Sakshi Piplani, Puneet Singh, David A. Winkler, Nikolai Petrovsky

**Affiliations:** 1 Vaxine Pty Ltd., 11 Walkley Avenue, Adelaide 5046, Australia; sakshi.piplani@flinders.edu.au (S.P.); puneet.singh@flinders.edu.au (P.S.); 2 Biochemistry and Chemistry Department, La Trobe University, Kingsbury Drive, Melbourne 3086, Australia; d.winkler@latrobe.edu.au; 3 Monash Institute of Pharmaceutical Sciences, Monash University, Parkville 3052, Australia; 4 School of Pharmacy, University of Nottingham, Nottingham NG7 2RD, UK; 5 Department of Diabetes and Endocrinology, Flinders Medical Centre, Flinders University, 1 Flinders Drive, Adelaide 5042, Australia

**Keywords:** SARS-CoV-2, COVID-19, helicase, molecular docking, molecular dynamics, drug repurposing, antivirals

## Abstract

Repurposing of existing drugs is a rapid way to find potential new treatments for SARS-CoV-2. Here, we applied a virtual screening approach using Autodock Vina and molecular dynamic simulation in tandem to screen and calculate binding energies of repurposed drugs against the SARS-CoV-2 helicase protein (non-structural protein nsp13). Amongst the top hits from our study were antivirals, antihistamines, and antipsychotics, plus a range of other drugs. Approximately 30% of our top 87 hits had published evidence indicating in vivo or in vitro SARS-CoV-2 activity. Top hits not previously reported to have SARS-CoV-2 activity included the antiviral agents, cabotegravir and RSV-604; the NK1 antagonist, aprepitant; the trypanocidal drug, aminoquinuride; the analgesic, antrafenine; the anticancer intercalator, epirubicin; the antihistamine, fexofenadine; and the anticoagulant, dicoumarol. These hits from our in silico SARS-CoV-2 helicase screen warrant further testing as potential COVID-19 treatments.

## 1. Introduction

The global COVID-19 pandemic continues to wreak economic and social havoc globally, with at least 330 million infections and 5.6 million deaths globally (January 2022). Effective vaccines and drug therapies are essential to bringing the pandemic to an end. This global challenge has seen an unprecedented and intense focus on coronavirus research, resulting in the development of vaccines in impressively short times. Similarly, empirical and limited rational selection of drugs such as remdesivir and dexamethasone provided early drug treatments that limited morbidity and mortality. However, more effective drugs are still required to treat COVID-19 and other coronavirus diseases, such as SARS and MERS, as well as new viruses that may emerge in the future.

Structural biologists have successfully characterized SARS-CoV-2 proteins that represent viable drug targets for structure-based computational design of new drugs, and for rapid repurposing of existing drugs for treatment of COVID-19. There has been extensive research into drugs that might interfere in the SARS-CoV-2 spike protein’s interaction with its cognate human receptor, angiotensin-converting enzyme 2 (ACE2). Other heavily studied targets are the viral 3CL main protease (M^pro^, nsp5), PL protease (PL^pro^, nsp3), and RNA-dependent RNA polymerase (RdRp, major protein nsp12) [1,2,3,4,5,6,7]. However, less attention has been paid to other potential target proteins such as the SARS-CoV-2 helicase, the focus of the current study.

Time is critical when developing vaccines or drug treatments; new drugs typically take many years to reach the clinic. Repurposing existing drugs, clinical trial candidates, and approved natural products that have been ingested by human beings and whose toxicity, pharmacokinetics, and metabolism are already well-understood is a rational and rapid way to find effective therapies during a pandemic [8]. Repurposing can be done by high-throughput in vitro assays, in vivo studies in animals, and computational drug design methods. Several teams have undertaken wet-lab screening of existing drugs against viral targets or viral assays, but none have achieved sufficient high throughput to allow >10,000 candidates to be assessed. Computational screening can be performed easily on large numbers of molecules, with relative binding affinities allowing ranking of the candidates for focused in vitro and in vivo testing followed by human antiviral trials, with minimal delay [9,10].

The SARS-CoV-2 helicase (non-structural protein nsp13) has been studied less but has considerable potential for the discovery of drugs against SARS-CoV-2. Of the 16 known CoV nsp proteins, the helicase is essential for viral replication and, not surprisingly, it has the highest sequence conservation across the CoV family [11]. As such, this vital enzyme represents a promising target for anti-CoV drug development as drugs targeting it have the potential to be active against all SARS-CoV-2 strains [12].

The helicase contains 601 amino acids and is part of the superfamily 1B, highly conserved within all coronaviruses. Helicases can have either 3′–5′ (SF1A subfamily) or 5′–3′ (SF1B subfamily) translocation polarity, defined as the direction (characterized as 5′→3′ or 3′→5′) of helicase movement on the DNA/RNA single strand along which it is moving [13,14,15,16,17]. The SARS-CoV-2 helicase is a critical enzyme for viral replication as it initiates the first step of the RNA cap synthesis that is essential to protect the virus from innate immune attack, stabilize it, and ensure its translation. We previously developed an in silico screening protocol that was used to identify drug repurposing candidates for SARS-CoV-2 M^pro^ and RdRp [5,6,7]. The utility of this approach was established by the large numbers of predicted candidates that had experimentally validated activity against SARS-CoV-2 and/or the specific target proteins.

Here, we describe a comprehensive, combined molecular docking and molecular dynamics (MD) study of registered drugs, drug candidates, and approved natural products against the SARS-CoV-2 helicase. We identify the most promising drug candidates for repurposing and validate many of the computational predictions using experimental data from the scientific literature.

## 2. Results and Discussion

**Figure 1:**
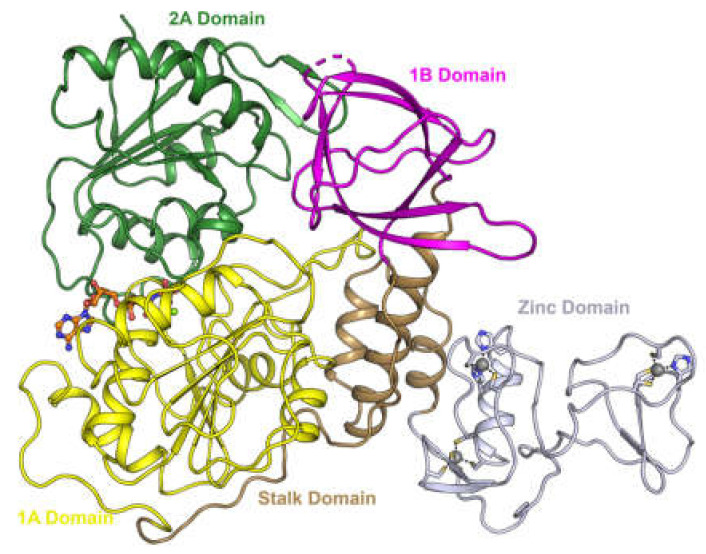
Domain structure of SARS-CoV-2 helicase. Adapted with permission from Ref. [18]. Creative Commons Attribution 4.0 International License (http://creativecommons.org/licenses/by/4.0/).

The helicase is a protein of 601 amino acids and has a shape of a triangular pyramid that is divided into five sections, a zinc-binding domain (ZBD) which is attached to two Rec-A domains (Rec1A and Rec2A) and a Rec1B domain via a stalk domain (Figure 1).

**Figure 2:**
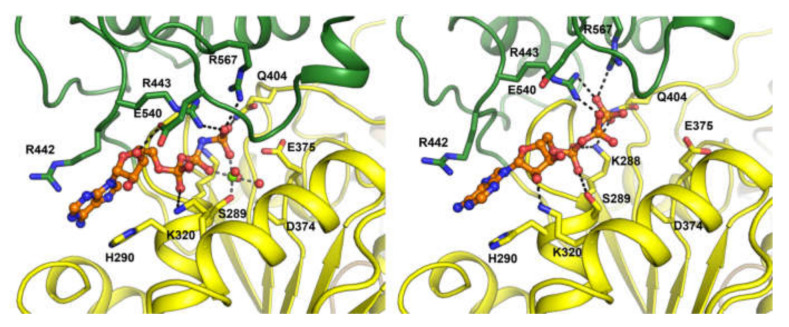
Key active ATP site residues for helicase bound to the AMP–PNP Mg2+ complex (**left**) and the AMP–PNP complex (**right**). Adapted with permission from Ref. [18]. Creative Commons Attribution 4.0 International License (http://creativecommons.org/licenses/by/4.0/).

The hydrolytic activity is attributed to six key residues (Lys288, Ser289, Asp374, Glu375, Gln404, Arg567) found within the cleft between the 1A and 2A domains at the base. These residues are located at the active site of SARS-CoV-2 helicase enzyme (Figure 2). The ATP binding pocket of helicase has a volume of 325 Å^3^ and an area of 420Å^2^ and is distinct to the RNA binding site.

In silico methods were used for calculating the relative binding affinities of drugs for the helicase ATP binding pocket. Autodock Vina docking followed by MD simulation of the top candidates was used to improve predictions of relative binding affinities compared to docking alone, with significant improvement in protein–ligand docking results by subsequent high-throughput MD simulations having been shown by others [19].

The MMPBSA and thermodynamic scores for the top 87 hits in our screen correlated strongly (r^2^ = 0.85). Given that many of the top-ranked molecules are conformationally flexible, binding energy penalties due to ligand entropy are likely to be significant. A substantial correlation was found between the Vina scores and the binding energies from MMPBSA and the thermodynamic cycle, important because of the different ways these algorithms treat ligand entropy [20].

The twenty molecules with the best helicase binding affinity are summarized in Table 1. The compounds can be broadly characterized as containing one or more hydrophobic aromatic moieties linked to another polycyclic moiety containing hydrogen bond donors or acceptors. The top-ranked molecules come from diverse drug classes, with antiviral agents making up 25% of the hits and antihistamines and antipsychotics also being well-represented.

The remaining high binding hits included drugs and natural products used to treat a diverse range of afflictions including cancers, infections, coagulation disorders, and hypertension. Several of the top hits from our screen had experimental their SARS-CoV-2 activity determined, as do ~30% of the 87 shortlisted compounds from our docking studies (Supplementary Table S1).

The calculated binding energies of the top-scoring antiviral drugs, dolutegravir, cabotegravir, bictegravir, tipranavir, and RSV-604 are similar (in the range of −38.8 to −42.2 kcal/mol by MMPBSA and −36.2 to −45.9 kcal/mol by thermodynamic cycle). Some of the highest-ranked antiviral agents had also been identified as good binders in other in silico docking studies, providing a degree of validation that our computational methods are appropriate and are yielding similar results to the other published studies for these antiviral drugs.

**Figure 3:**
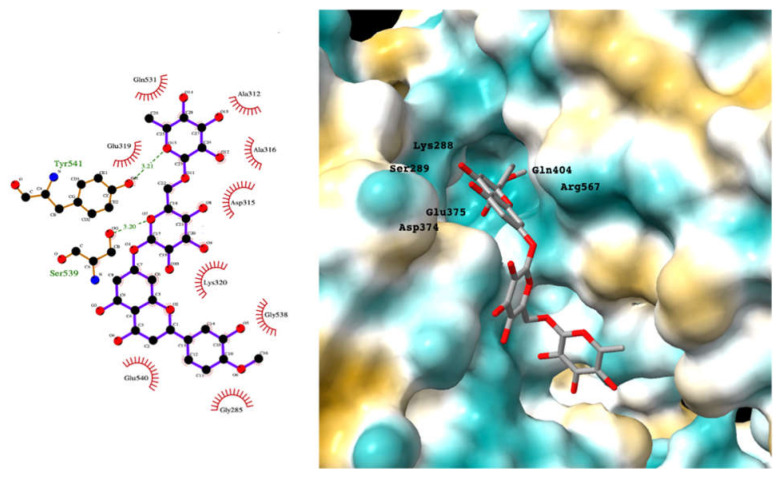
LigPlot (**left**) shows the key active site residues interacting with hesperidin. The molecular model (**right**) shows the binding of hesperidin to the active site cleft of the helicase. The molecular surface denotes hydrophobicity of the pockets (blue hydrophilic, yellow/brown hydrophobic). Positions of key binding site residues are labeled.

**Figure 4:**
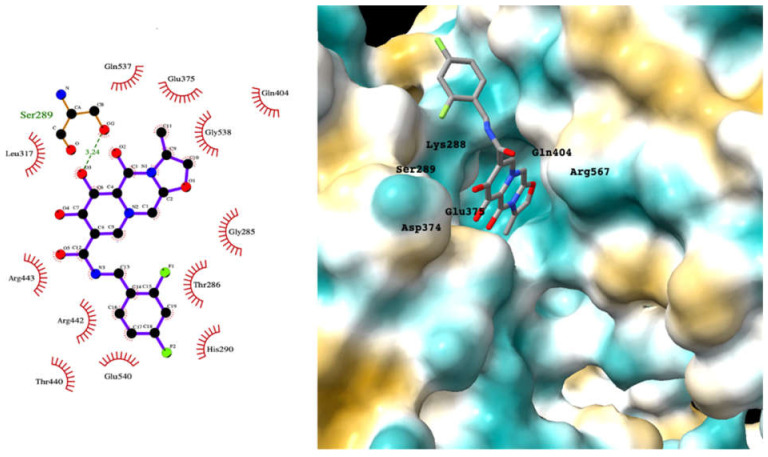
LigPlot (**left**) shows the key active site residues interacting with cabotegravir. The molecular model (**right**) shows the binding of cabotegravir to the active site cleft of the helicase. The tricyclic ring moiety is deeply embedded in a hydrophilic pocket. The molecular surface denotes hydrophobicity of the pockets (blue hydrophilic, yellow/brown hydrophobic). Positions of key binding site residues are labeled.

**Figure 5:**
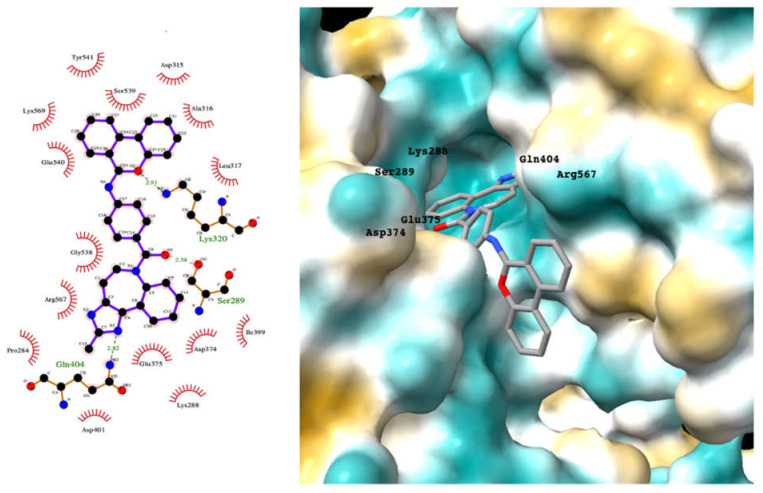
LigPlot (**left**) shows the key active site residues interacting with canivaptan. The molecular model (**right**) shows the binding of canivaptan to the active site cleft of the helicase. The tricyclic ring moiety is deeply embedded in a hydrophilic pocket. The molecular surface denotes hydrophobicity of the pockets (blue hydrophilic, yellow/brown hydrophobic). Positions of key binding site residues are labeled.

**Figure 6:**
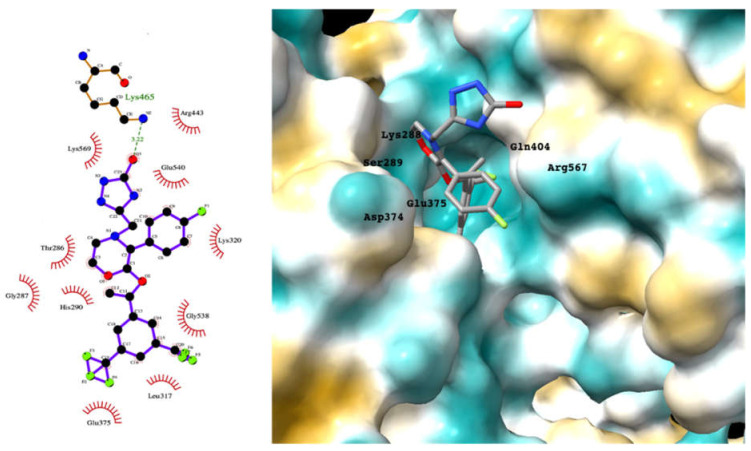
LigPlot (**left**) shows the key active site residues interacting with aprepitant. The molecular model (**right**) shows the binding of aprepitant to the active site cleft of the helicase. The molecular surface denotes hydrophobicity of the pockets (blue hydrophilic, yellow/brown hydrophobic). Positions of key binding site residues are labeled. Aprepitant (NK1 antagonist) buries the morpholino ether moiety in the hydrophilic pocket and the hydrophobic bis trifluoromethyl substituted ring even deeper into this pocket. The environment mismatch is compensated by the favorable π-stacking and π-cation interactions referred to above.

Although the main aim of this study was to show that our computational methods are useful for rapidly identifying repurposed drugs likely to exhibit SARS-CoV-2 activities, we have also analyzed the binding of key repurposed drugs (Table 1) to the helicase active ATP binding site. Figure 3, Figure 4, Figure 5 and Figure 6 show LigPlot diagrams of the main interactions between four of the drugs and the ATP binding site. They elucidate how the drugs bind to the active ATP site of the helicase enzyme. Supplementary Figure S2 shows a superimposition of the drugs with the most favorable binding energies in the active ATP site of the helicase.

For hesperidin (Figure 3), four of the five rings are buried deep in the ATP binding pocket, with the hydrophilic terminal sugar ring being exposed to the solvent. There are networks of hydrogens bond between the active site residues and the donor and acceptor groups on most repurposed drugs, especially for aminoquinuride and rutin.

Supplementary Table S2 lists the key interactions between the top screening hits and the active ATP site of the helicase. The 6 residues in helicase binding pocket, Lys288, Ser289, Asp374, Glu375, Gln404, and Arg567, are crucial for ATP hydrolysis, and all screened drugs interacted strongly with these residues. These molecular-level interactions involved in binding the drugs at ATP binding site of the SARS-CoV-2 helicase enzyme were investigated to decipher the key chemical forces crucial for intermolecular binding and stability of complexes. Cabetogravir, dicoumarol, fexofenadine, epirubicin, antrafenine, aminoquinuride, aprepitant, and RSV-604 all formed strong hydrogen bonds with residues in the ATP-binding site at Rec1A domain. Aprepitant also formed π-stacking (Try541) and π-cation (Lys320) interactions with nsp-13. Antrafenine and aprepitant form halogen bonds with Ile399 and Asp374 respectively.

For the antiviral integrase drug, carbogravir (Figure 4), the tricyclic ring is buried deep within the active site of the helicase. Again, the abundant hydrogen bond donors and acceptors on the polycyclic moiety form hydrogen bonds with the binding site residues of the helicase.

The vasopressin inhibitor, canivaptan (Figure 5), buries its hydrophilic nitrogen-rich heterocyclic rings deep within the ATP binding cleft, forming a range of hydrogen bonds with active site residues.

### 2.1. Other Computational Studies Identifying Compounds amongst Our Top 20 Candidates

The only prior computational study that proposed that our top-ranked drug candidate dolutegravir may inhibit SARS-CoV-2 helicase involved a deep learning model [21]. This study suggested that dolutegravir also inhibits several other viral targets. Indu et al. also used MD studies and Autodock Vina to identify dolutegravir as a potential inhibitor of SARS-CoV-2 M^pro^ and RdRp [22]. If these activities of dolutegravir are subsequently confirmed, its ability to hit several viral targets simultaneously may make it particularly effective for treating COVID-19 patients. For example, such multitarget drug approaches have been very effective in controlling HIV infections.

**Figure 7:**
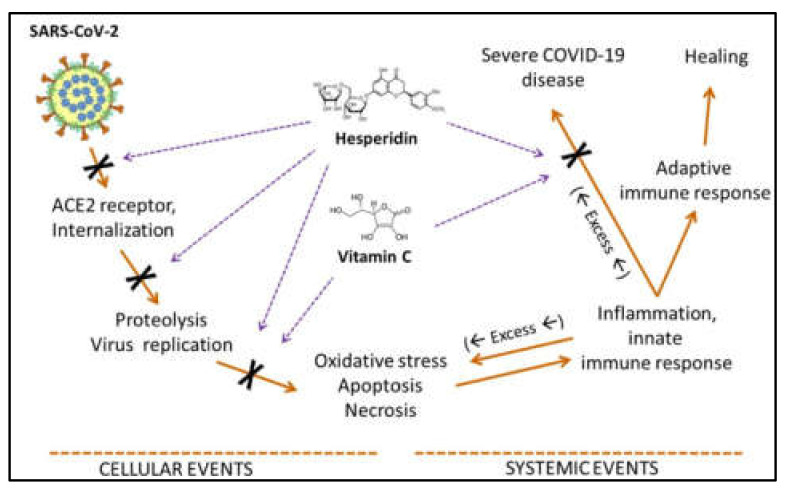
Multiple effects of hesperidin (coupled with ascorbic acid) on SARS-CoV-2 entry and replication, and systemic inflammation. Creative Commons Attribution (CC BY) license from Bellavite and Donzelli [25].

Computational docking experiments by Adem et al. and Mosquer-Yuqui et al. identified hesperidin as a potential treatment for COVID-19 [23,24]. Adem et al. used Molegro Virtual Docker 7 to analyze 80 flavonoid compounds binding to M^pro^ and found hesperidin had the highest predicted binding affinity [23]. Similarly, Mosquer-Yuqui et al. screened 92 phytochemicals from Andean medicinal plants against SARS-CoV-2 M^pro^ and RNA-dependent RNA polymerase (RdRp) using molecular docking [24]. Unlike Adem et al., they subsequently simulated the interactions of the top-ranked natural products, including hesperidin, using GROMACS MD. Hesperidin was also suggested as an antiviral candidate by other studies [25,26,27]. For example, Meneguzzo et al. reported that hesperidin had a high binding affinity to ACE2 and could block SARS-CoV-2 entry via this receptor (Figure 7), outperforming drugs already recommended for human COVID-19 trials [27].

There are no prior reports of cabotegravir activity against the SARS-CoV-2 helicase, although Petersen et al. predicted that cabotegravir might bind to M^pro^ using a combination of molecular docking and MD calculations [28]. Similarly, no studies have reported conivaptan as having potential helicase activity, although Gul et al. predicted conivaptan had activity against SARS-CoV-2 RdRp [29]. Potential conivaptan binding to the SARS-CoV-2 nsp9 replicase was found by Chandel et al. using a combination of Autodock screening followed by MD simulations [30]. Several other recent computational studies have also reported potential binding of conivaptan to various SARS-CoV-2 targets (see summary in Piplani et al.) [6,7].

Two prior studies predicted aprepitant (Emend) to be a helicase inhibitor. White et al. used Autodock Vina followed by MD simulations to identify aprepitant as having potential helicase activity [11]. Borgio et al. also identified aprepitant as having potential helicase activity using MOE molecular docking and the MOE score or GBVI/WSA binding free energies [31].

Potential helicase activity of bictegravir has not been reported previously. However several computational docking studies identified bictegravir as having potential activity against the SARS-CoV-2 2′-O-ribose methyltransferase (2′-O-MTase) [32], the spike glycoprotein [33], and M^pro^ [28].

Manidipine was predicted to be a promising binder to M^pro^ in a virtual screen using Glide SP, AutoDock Vina, and two protocols with AutoDock 4.2 followed by MD simulation using Gromacs [34].

Tipranavir has been identified as a potential SARS-CoV-2 inhibitor in in vitro screens. No other computational studies have predicted tipranavir to be a helicase inhibitor. Kumar et al. reported potential activity against M^pro^ using docking with MD simulation [35], whereas Gul et al., using a similar approach, suggested tipranavir would have activity against both M^pro^ and RdRp [36]. Autodock Vina was also used by Mohamed et al. to identify tipranavir as a potential inhibitor of SARS-CoV-2 PL^pro^ [10].

Activity of antrafenine against SARS-CoV-2 helicase was predicted by Wu et al. (Supplementary Table S1) using a homology model and the ICM 3.7.3 modeling software [37]. Mevada et al. used Autodock Vina to screen drug candidates against SARS-CoV-2, including against the helicase, and antrafenine was shown to be potentially active against the helicase and many other targets, including the viral spike protein [38]. However, as no post-docking MD simulations were conducted on the lead molecules, the results should be viewed with caution. Cozac et al. used machine learning and Autodock Vina calculations to predict antrafenine as an inhibitor of RdRp [39].

In an earlier computational study, we identified RSV-604 as a potential SARS-CoV-2 M^pro^ inhibitor using Autodock Vina followed by MD simulation of the lead molecules in the active site of the protein [5]. Although no previous studies have predicted epirubicin to have activity against the SARS-CoV-2 helicase it has been identified as an inhibitor of chromodomain-helicase-DNA-binding protein 1 and HCV helicase [40,41]. No prior studies have reported activity of fluspirilene against the SARS-CoV-2 helicase, although it has been predicted to have activity against the SARS-CoV-2 nsp9 replicase [42]. Tam et al. reported it to have M^pro^ activity, and the DG of binding calculated by Autodock Vina correlated well with experimental DG of binding from the experimental IC_50_ values of a range of putative antiviral agents [43].

Fexofenadine was predicted to have good M^pro^ activity by Autodock Vina and to be a moderate binder by Autodock [44,45]. Astemizole was also predicted to bind well to the SARS-CoV-2 spike protein by the PLANTS algorithm. It was in the top 2% of molecules that were rescored using MD (Amber and the Nwat-MMGBSA method) [46,47]. It was also predicted to bind to the viral RdRp with high affinity using Autodock Vina [48].

Our earlier studies predicted sertindole to be a strong binder to SARS-CoV-2 RdRp [6,7], and the activity of sertindole against M^pro^ also was later reported by Vatensever et al. [44].

### 2.2. Experimental Validation of Computational Predictions for Top 20 Repurposed Candidates

Xie et al. reported a nanoluciferase assay in Vero E6 cells for SARS-CoV-2 that returned an EC_50_ > 10 µM and CC_50_ > 50 µM for bictegravir [49]. This assay also found that remdesivir and chloroquine were highly active, although responses in human clinical trials have been less than impressive. However, remdesivir has received emergency use authorization for the treatment of COVID-19 infections.

**Figure 8:**
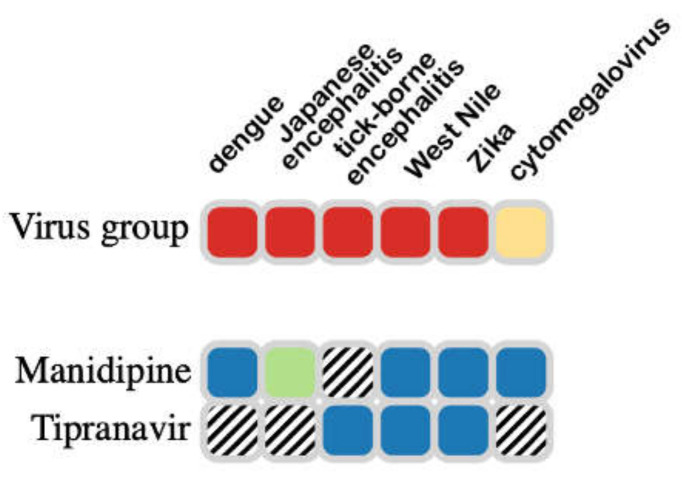
Confirmation of experimental antiviral activity for two of the lead hits, shown to have antiviral activity in (cell assays (blue) or animal models (green). From https://drugvirus.info/ (accessed on 1 April 2022).

Manidipine has relatively broad-spectrum antiviral activity (see Figure 8), with in vitro IC_50_ values of 10 µM against SARS-CoV-2 M^pro^ and 14 µM against PL^pro^. It also exhibited antiviral activity against the SARS-CoV-2 virus with EC_50_ of 15 ± 1 µM in a plaque reduction assay [50]. Ghahremanpour et al. measured the activity of manidipine in a kinetic M^pro^ assay as 4.8 μM, and Pickard et al. measured its activity in HUH7 cells (IC_50_ = 2 µM) and Vero cells (IC_50_ = 7.5 µM) [51].

As summarized in Figure 8, tipranavir also exhibits a relatively broad spectrum of antiviral activity. It was shown to inhibit replication of SARS-CoV-2 in VeroE6 cells, but the SI was relatively low (EC_50_ = 13 μM, CC_50_ = 77 μM, SI = 6) [52]. There are no experimental studies showing that pimozide inhibits the SARS-CoV-2 helicase. Vatansever et al. identified pimozide as a basic molecule that raises endosomal pH to interfere with SARS-CoV-2 entry into the human cell host, and measured an IC_50_ against M^pro^ of 42 ± 2 µM [44].

There is no published computational or experimental binding data for rutin binding to SARS-CoV-2 helicase, although Huynh et al., amongst several others, reported that MD calculations predicted rutin binding to M^pro^ [53]. Aprepitant, which was predicted by our study to have activity against the helicase, has been shown to be effective in treating severe to critical COVID-19 patients in combination with dexamethasone (https://clinicaltrials.gov/ (accessed on 1 April 2022), NCT04468646) [54].

There are no reports in the literature of dicoumerol having activity against SARS-CoV-2, only a single report predicting binding to M^pro^ by Balakrishnan et al. [55]. Fluspirilene activity against SARS-CoV-2 was reported by Weston et al. They measured the IC_50_ as 3.1 µM, CC_50_ as 30.3 µM and SI = 10 in Vero E6 cells [56]. This mirrors the activity of fluspirilene against MERS-CoV and SARS-CoV in Vero E6 cells of 7.5 µM and 6.0 µM, respectively [57].

Doramapimod was reported to have an IC_50_ of 10 µM against SARS-CoV-2 in MRC5-ACE2 cells, and showed synergism with remdesivir in killing the virus in vitro [58]. Astemizole was reported to have an EC_50_ of 1 µM SARS-CoV-2 in Vero E6 cells, together with EC_50_ values against MERS-CoV and SARS-CoV of 4.9 µM and 5.5 µM, respectively [57,59].

### 2.3. Experimental Validation of Predictions

Apart from the top 20 drug-repurposing candidates with the highest predicted binding affinities to SARS-CoV-2 helicase, a significant number of our other hits listed in Supplementary Table S1 also have published experimental validation of SARS-CoV-2 activity. Indeed, it is noteworthy that almost 30% of the drugs in our top 87 drug-repurposing candidates have experimentally confirmed SARS-CoV-2 activity in vitro. While in vitro activity does not mean that all these drugs are operating by inhibiting the virus helicase or will have activity against the virus in vivo, this data suggests that our computational screening method identified candidates that are enriched in compounds active against the virus. Our study suggests there is value in using in vitro assays to further screen the compounds in Supplementary Table S1 that have not yet been tested to potentially yield additional existing drugs with unrecognized activity against SARS-CoV-2. As they are already approved drugs, any promising candidates can be rapidly advanced to human trials.

An increasing amount of data is becoming available from high throughput physical screens of helicase active compounds that may hopefully validate additional of our hits. For example, a group developed a novel fluorescence resonance energy transfer-based strand displacement assay for monitoring SARS-CoV-2 helicase activity and used this assay to screen a custom chemical library of over 5000 approved and investigational compounds for novel helicase inhibitors, identifying three novel compounds and confirming suramin-like compounds as helicase inhibitors [60]. Another group established biochemical assays for SARS-CoV-2 nps13-associated enzyme activities on RNA unwinding and 5′-triphosphatase activity and used these to screen a small in-house library of natural compounds identifying myricetin, quercetin, kaempferol, and flavanone as inhibitors of RNA unwinding activity and licoflavone C as an inhibitor of both helicase activities [61]. A compound based on a 2-phenylquinoline scaffold was shown to have potent in vitro activity against SARS-CoV-2 helicase [62].

### 2.4. Other Helicase in Silico Studies

Since the initial conduct of our study, several additional in silico studies of SARS-CoV-2 helicase activity have been reported that will help refine future in silico drug screening efforts. Berta et al., performed microsecond long MD simulations on the SARS-CoV-2 helicase complex to characterize the enzyme motions and identify potential allosteric binding sites [63]. Chen et al., also used prolonged MD simulations to analyze Cryo EM structures of the helicase and show that the helicase can have four distinct conformational states to help explain its multiple actions [64].

Amongst other helicase drug screening studies, a docking study screened nucleoside analogs for helicase activity and identified pritelivir as a potential drug candidate [65]. Another molecular docking and MD simulation study identified potential natural product inhibitors of helicase targeting the ATP-binding site which included picrasidine-N and -M, epiexcelsin, isorhoeadine, and euphorbetin [66]. Another study used fragment screening to identify possible druggable pockets on the Nsp13 helicase, identifying a favorable allosteric site on the N-terminal zinc binding domain that is a Nsp8:Nsp13 protein–protein interaction site [67]. Yet another in silico study screened the Medicinal Plant Database for Drug Design database for helicase binders [68]. A homology model that couples published electron density with molecular-dynamics-based structural refinements was used to generate models of the helicase in its apo- and ATP/RNA-bound conformations with these models; it was then used to screen ∼970,000 compounds against the ATP-binding site and identified cepharanthine and lumacaftor as potential inhibitors [11]. Another study used simulations of mutant helicase proteins to identify drugs retaining activity against both the wildtype and mutant proteins, identifying cangrelor, fludarabine, folic acid, and polydatin as the most promising candidates [69].

### 2.5. Potential Study Limitations

In silico predictions always ultimately require experimental validation, which we have attempted to achieve indirectly by screening the literature for evidence that the hits from our helicase predictions have elsewhere been shown to have activity against SARS-CoV-2, identifying supporting data for at least 30% of our top hits. We used the Cryo EM structure 6XEZ for our docking calculations, which had a reported resolution of 2.9 Å but had significantly lower resolution around the helicase domains and contained a number of modeling errors and missing sidechain residues, even around the helicase active site (carbonyl of T286, sidechains of R442 and R443), due to the poor-quality crystal structure (6JYT) used as a template for the Cryo EM reconstruction. More recent structures have been released that provide additional important information on the helicase and the complexes it forms, including PDB structure 7NNO of a monomer binding an ATP analogue ANP and structures of the helicase complex [70]. These new, more-accurate EM structures, plus the MD analyses of Berta et al. and Chen et al., will allow future helicase modeling studies such as ours to be repeated with even more precision. This emphasizes the importance of good crystal structures for informing in silico high-throughput screening; new structural data is becoming available all the time. Another potential limitation of our study is the relatively short simulation time for the MD analyses, a limitation imposed by the computing power needed to run large numbers of simulations. While other studies have used longer simulation times, this is generally because they have been focused on just a single or a few structures, in which case this is logistically possible. The nsp13 helicase protein is very flexible and the ATP binding site undergoes relatively large changes in volume as the RecA domains move relative to each other; this means that the system may take significantly longer than 20 ns for RMSDs to stabilize.

However, regardless of their limitations, and while predictions will always require experimental validation, the increasing accuracy and speed of in silico drug screening methods means that they are likely to be increasingly used for drug screening by both academia and industry, with their speed being particularly attractive for their use in pandemic drug screening.

## 3. Methods and Materials

### 3.1. Protein Structure Preparation and Grid Preparation

The Protein Data Bank (PDB) file of the SARS-CoV-2 helicase 6XEZ (https://www.rcsb.org/structure/6XEZ) with a reported resolution of 2.90Å was downloaded (last accessed on 24 May 2022). Protein preparation, removal of non-essential and non-bridging water molecules, addition of hydrogen atoms and missing residues, and loops for docking studies were performed using UCSF Chimera package (https://www.cgl.ucsf.edu/chimera/, accessed on 24 May 2022) [71].

### 3.2. Screening Databases

A total of 11,875 drugs were retrieved from DrugBank database (FDA approved) in April 2020. The drugs were downloaded in .sdf format and converted to .pdbqt format using Raccoon [72].

### 3.3. Docking Methodology

All unique small-molecule drug structures were docked against the helicase protein structure using the AutoDock Vina (version 1.1.3) package [72]. AutoDock Vina employs a gradient-based conformational search approach and an energy-based empirical scoring function. AutoDock Vina is also flexible, easily scripted, extensively validated in many published studies with a variety of proteins and ligands, and takes advantage of large multi-CPU machines to run many calculations in parallel. The code has also been employed very successfully to dock millions of small-molecule drug candidates into a series of protein targets to discover new potent drug leads. The package includes useful scripts for generating modified .pdb files required for grid calculations and for setting up the grid calculations around each protein automatically. AutoDock Tools (ADT) was used to prepare the required files for Autodock Vina [72]. Non-essential heteroatoms, unnecessary protein chains or substructures (if any), and water molecules were removed, and non-polar hydrogen atoms were added to the protein structure, which was converted to .pdbqt format. Binding pockets were predicted using castp (http://sts.bioe.uic.edu/castp, accessed on 24 May 2022) AutoDock Vina requires the removal of hydrogens, the addition of polar hydrogens, setting of the correct atom types, and calculation of atom charges compatible with the AutoGrid code. The algorithm generates a grid around each protein and calculates the interaction energy of a probe noble gas atom at each grid position outside and within the internal cavities of the protein. The grid size used was 137.6 × 210.1 × 135.1 Å. Grid resolution was set to 1 Å, the maximum number of binding modes to output was fixed at 10, and the exhaustiveness level (controlling the number of independent runs performed) was set at 8. The docking calculations employed a genetic algorithm to optimize the binding conformations of the ligands during docking to the helicase site. Drugs were docked individually to the active site of the helicase with the grid coordinates (grid center) and grid boxes of appropriate sizes generated by the bash script vina_screen.sh (Supplementary Table S4). The top-scoring compounds were identified with the Python script ‘script1.py’ (Supplementary Table S4) and subjected to molecular dynamics simulation. The docked structures were analyzed using UCSF Chimera and LigPlot+ software to illustrate hydrogen bond and hydrophobic interactions [71,73]. The 87 drugs from Drugbank database with the most favorable helicase binding energies were selected (see Supplementary Materials). Molecular dynamics studies were subsequently conducted on this set of 87 compounds.

### 3.4. Molecular Dynamics Simulations

The top-screened compound complexes with the helicase were minimized with CHARMm force field. The topology files of the ligands were prepared from Swissparam (http://www.swissparam.ch/, accessed on 24 May 2022) and minimized in Gromacs2020 (http://www.gromacs.org/, accessed on 24 May 2022) [74,75]. Docked complexes of ligands and the helicase protein were used as starting geometries for MD simulations. Simulations were carried out using the GPU accelerated version of the program with the CHARMm force field I periodic boundary conditions in the ORACLE server. Docked complexes were immersed in a truncated octahedron box of TIP3P water molecules. The solvated box was further neutralized with Na+ or Cl− counter ions using the tleap program. Particle Mesh Ewald (PME) was employed to calculate the long-range electrostatic interactions. The cut-off distance for the long-range van der Waals (VDW) energy term was 12.0 Å. The whole system was minimized without any restraint. The complexes were subjected to 2500 cycles of steepest descent minimization followed by 5000 cycles of conjugate gradient minimization. After system optimization, the MD simulations were initiated by gradually heating each system in the NVT ensemble from 0 to 300 K for 50 ps using a Langevin thermostat with a coupling coefficient of 1.0/ps and with a force constant of 2.0 kcal/mol·Å2 on the complex. Finally, a production run of 20 ns of MD simulation was performed under a constant temperature of 300 K in the NPT ensemble with periodic boundary conditions for each system. During the MD procedure, the SHAKE algorithm was used to constrain all covalent bonds involving hydrogen atoms. The time step was set to 2 fs. The structural stability of the complex was monitored by the RMSD and RMSF values of the backbone atoms of the entire protein. Calculations were also performed for up to 100 ns on a few compounds to ensure that 20 ns was sufficiently long for convergence. Estimated uncertainties in the binding energies were < 1 kcal/mol.

The binding free energies of the protein–ligand complexes were evaluated in two ways. The traditional method is to calculate the energies of solvated SARS-CoV-2 helicase and small-molecule ligands and that of the bound complex and derive the binding energy by subtraction.

ΔG (binding, aq) = ΔG (complex, aq) − (ΔG (protein, aq) + ΔG (ligand, aq)(1)

We also calculated binding energies using the ‘molecular mechanics/Poisson–Boltzmann surface area’ (MM/PBSA) tool in GROMACS that is derived from the nonbonded interaction energies of the complex. The method is also a widely used method for binding free energy calculations.

MMPBSA calculations were conducted with GMXPBSA 2.1, a suite based on Bash/Perl scripts for streamlining MMPBSA calculations on structural ensembles derived from GROMACS trajectories and for automatically calculating binding free energies for protein–protein or ligand–protein interactions [76]. GMXPBSA 2.1 calculates diverse MMPBSA energy contributions from molecular mechanics (MM) and electrostatic contribution to solvation (PB) and non-polar contribution to solvation (SA). This tool combines the capability of MD simulations (GROMACS) and the Poisson-Boltzmann equation (APBS) for calculating solvation energy (Baker et., 2001). The g_mmpbsa tool in GROMACS was used after molecular dynamics simulations and the output files obtained were used to post-process binding free energies by the single-trajectory MMPBSA method. In the current study, we used 100 frames at equal distances from 20ns trajectory files.

Specifically, for a non-covalent binding interaction in the aqueous phase, the binding free energy, ΔG (bind,aq), is:

ΔG (bind,aq) = ΔG (bind,vac) + ΔG (bind,solv)(2)

where ΔG (bind,vac) is the binding free energy in vacuum, and ΔG(bind,solv) is the solvation free energy change upon binding:

ΔG (bind,solv) = ΔG (R:L, solv) − ΔG (R,solv) − ΔG (L,solv)(3)

where ΔG (R:L,solv), ΔG (R,solv) and ΔG (L,solv) are solvation free energies of complex, receptor and ligand, respectively.

## 4. Conclusions

Here, we show that the combination of advanced molecular docking algorithms with molecular dynamics simulations can reliably identify existing known drugs with potential activity against the SARS-CoV-2 helicase. These candidates, if confirmed, could then be rapidly deployed to treat COVID-19 patients in clinical trials. The predictions of our computational studies have largely been validated by parallel experimental in vitro testing by other groups. Given the high speed with which potential COVID-19 drug candidates can be identified using computational methods, the approach is highly suited for rapidly identifying promising drugs, not just for the current pandemic, but for those outbreaks that will inevitably occur in the future.

## Figures and Tables

**Table 1:** Binding energies of top 20 hits (ranked by MMPBSA score) against SARS-CoV-2 helicase.

Database ID	Drug Name	Structure	DG_MMPBSA_ kcal/mol	DG_thermo_ kcal/mol
DB08930	Dolutegravir (antiretroviral)	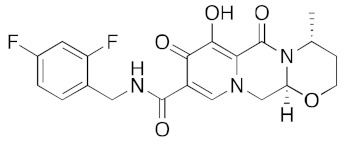	−42.2	−45.9
DB04703	Hesperidin (citrus flavanone glycoside)	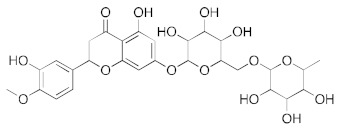	−41.2	−39.6
DB11751	Cabotegravir (antiviral integrase inhibitor)	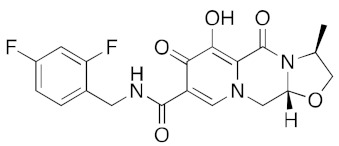	−40.8	−42.3
DB00872	Conivaptan (vasopressin inhibitor)	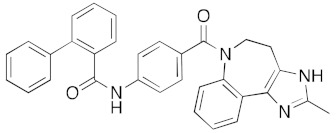	−40.2	−39.7
DB00673	Aprepitant (NK1 antagonist)	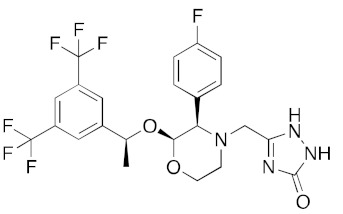	−40.1	−45.6
DB11799	Bictegravir (antiviral integrase inhibitor)	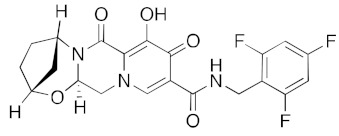	−39.8	−41.2
DB09238	Manidipine (Ca channel blocker, anti-hypertensive)	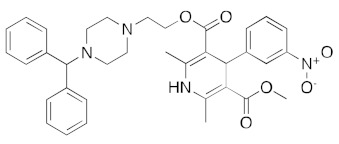	−39.8	−41.3
DB00932	Tipranavir (antiviral protease inhibitor)	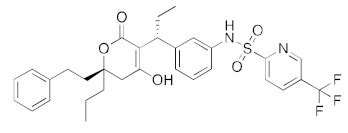	−39.7	−42.6
DB04452	Aminoquinuride (trypanocidal agent)	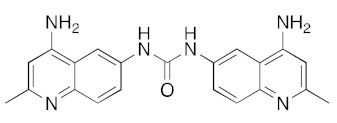	−39.6	−41.9
DB01419	Antrafenine (analgesic anti-inflammatory)	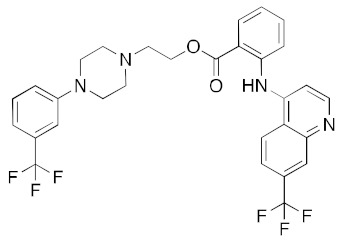	−39.1	−36.5
DB15197	RSV-604 (antiviral)	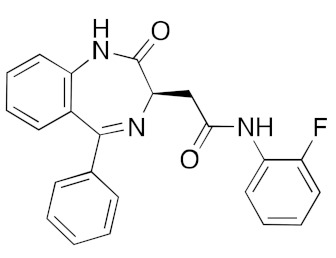	−38.8	−36.2
DB004445	Epirubicin (anticancer intercalator)	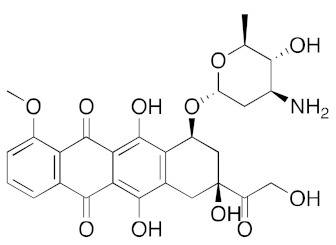	−38.7	−35.6
DB01100	Pimozide (antipsychotic)	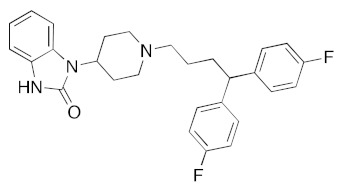	−38.4	−35.4
DB01698	Rutin (flavonol glycoside)	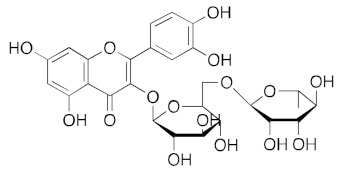	−38.3	−40.4
DB00266	Dicoumarol (anticoagulant)	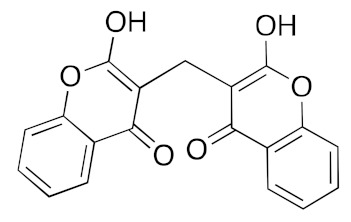	−37.6	−38.4
DB04842	Fluspirilene (antipsychotic)	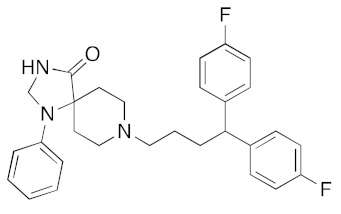	−36.8	−38.4
DB03044	Doramapimod (p38 MAP kinase inhibitor)	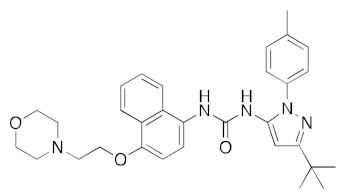	−36.2	−39.5
DB00950	Fexofenadine (antihistamine)	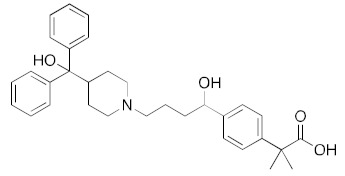	−35.5	−37.7
DB00637	Astemizole (antihistamine)	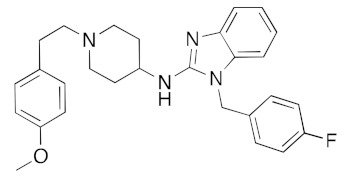	−35.4	−38.7
DB01100	Sertindole (antipsychotic)	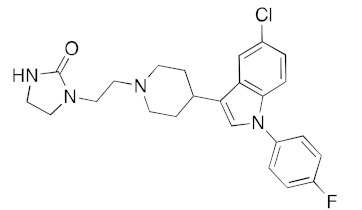	−34.6	−36.8

## Data Availability

Data will be deposited in the OPAL/figshare data repository at La Trobe University (https://opal.latrobe.edu.au/), and a DOI will be generated when this paper is published.

## Supplementary Materials

The following supporting information can be downloaded at: https://www.mdpi.com/article/10.3390/ijms23147704/s1. Refs. [77,78,79,80,81,82,83,84,85,86,87,88,89,90,91,92,93] are cited in Supplementary Materials.
