# Supplementary material for: Potential COVID-19 Therapies from Computational Repurposing of Drugs and Natural Products against the SARS-CoV-2 Helicase
Source: Int J Mol Sci. 2022 Jul 12;23(14):7704. doi: 10.3390/ijms23147704 (PMC9322913; doi:10.3390/ijms23147704)
Supplement: Supplementary file 1 [file ijms-23-07704-s001.zip › Helicase supplementary information.pdf]

Putative COVID-19 therapies from computational repurposing of drugs and natural products against the SARS-CoV-2 helicase

Sakshi Piplani [1], Puneet Singh [1], David A. Winkler [2,3,4] and Nikolai Petrovsky [1,5,*]

[1] Vaxine Pty Ltd., 11 Walkley Avenue, Adelaide 5046, Australia; sakshi.piplani@flinders.edu.au (S.P.); puneet.singh@flinders.edu.au (P.S.)
[2] Biochemistry and Chemistry Department, La Trobe University, Kingsbury Drive, Bundoora 3042, Australia; d.winkler@latrobe.edu.au
[3] Monash Institute of Pharmaceutical Sciences, Monash University, Parkville 3052, Australia
[4] School of Pharmacy, University of Nottingham, Nottingham NG7 2RD, UK
[5] Department of Diabetes and Endocrinology, Flinders Medical Centre, Flinders University, 1 Flinders Drive, Bedford Park 5042, Australia
* Correspondence: nikolai.petrovsky@flinders.edu.au

Supplementary information

Table S1. Binding scores and previously published SARS-Cov-2 data for 88 top ranked SARS-CoV-2 helicase hits.

No.	Drugbank ID	Name	ΔG_{MMPBSA} kcal/mol	ΔG_{thermo} kcal/mol	Experimental SARS-CoV-2 data
1	DB08930	Dolutegravir	-42.15	-45.87	SARS-CoV-2 activity in Vero cells EC_{50} = 22μM, CC_{50} >40μM.[1]
2	DB04703	Hesperidin	-41.23	-39.58	SARS-Cov-2 Mpro inhibition IC_{50} = 8.3 μM.[2]
3	DB11751	Cabotegravir	-40.77	-42.31	…
4	DB00872	Conivaptan	-40.20	-39.65	SARS-CoV-2 EC50 10μM CC_{50} 13μM in HEK-293T cells.[3] EC_{50} = 12.2 μM against HCoV-OC43.
5	DB00673	Aprepitant	-40.12	-45.58	…
6	DB11799	Bictegravir	-39.78	-41.22	Vero E6 cells for SARS-CoV-2 that returned an EC_{50}>10μM and CC_{50} >50μM for bictegravir.[4]
7	DB09238	Manidipine	-39.77	-41.25	IC_{50} 10μM against SARS-CoV-2 M^{pro} ; 14μM against PL^{pro}. Apparent SARS-CoV-2 EC_{50} = 15±1 μM in a plaque reduction assay.[5] et al. Kinetic M^{pro} IC_{50} = 4.8 μM. SARS-CoV-2 activity in in HUH7 cells (IC_{50}=2μM) and Vero cells (IC_{50}=7.5μM).[6]
8	DB00932	Tipranavir	-39.74	-42.56	Inhibits replication of SARS-CoV-2 in VeroE6 cells, but low SI (EC_{50} = 13 μM, CC_{50} = 77 μM, SI = 6).[7]
9	DB04452	Aminoquinuride	-39.58	-41.89	…
10	DB01419	Antrafenine	-39.14	-36.47	…
11	DB15197	RSV-604	-38.75	-36.21	…
12	DB004445	Epirubicin	-38.74	-35.62	…
13	DB01100	Pimozide	-38.36	-35.44	IC_{50} for SARS-CoV-2 M^{pro} = 42 ± 2μM.[8]
14	DB01698	Rutin	-38.32	-40.44	IC_{50}=32 μM for SARS-CoV-2 3CLpro.[9]

No.	Drugbank ID	Name	ΔG_{MMPBSA} kcal/mol	ΔG_{thermo} kcal/mol	Experimental SARS-CoV-2 data
15	DB00266	Dicoumarol	-37.56	-38.39	…
16	DB04842	Fluspirilene	-36.77	-38.38	IC_{50} = 3.1µM and CC_{50} = 30.3µM with SI=10 in Vero E6 cells.[10] In vitro activity in MERS-CoV and SARS-CoV-1 (SARS) = 7.5µM and 6.0µM respectively in Vero E6 cells.[11]
17	DB03044	Doramapimod	-36.21	-39.45	IC_{50} = 10µM against SARS-CoV-2 in MRC5-ACE2 cells. Useful synergism with remdesivir in killing the virus in vitro.[12]
18	DB00950	Fexofenadine	-35.47	-37.66	…
19	DB00637	Astemizole	-35.35	-38.70	EC_{50} = 1µM in Vero E6 cells infected with SARS-CoV-2.[13] EC_{50} values for MERS-CoV and SARS-CoV-1 (SARS) of 4.9µM and 5.5µM respectively.[11]
20	DB01100	Pimozide	-34.67	-38.21	SARS-CoV-2 Mpro IC_{50} = 42 µM.[8]
21	DB06144	Sertindole	-34.56	-36.77	…
22	DB12580	Tradipitant	-34.21	-32.11	Phase 3 clinical trial for COVID-19 NCT04326426.[14]
23	DB08881	Vemurafenib	-33.78	-30.25	SARS-CoV-2 in vitro inhibition in Vero cells, IC_{50} =7.0 µM and CC_{50} >50 µM.[15]
24	DB09295	Talniflumate	-33.22	-31.47	…
25	DB09048	Netupitant	-32.45	-30.74	…
26	DB11759	Penvonedistat	-32.44	-35.41	…
27	DB00966	Telmisartan	-32.31	-35.08	…
28	DB09298	Silibinin	-32.26	-30.41	…
29	DB11995	Avatrombopag	-31.52	-35.47	…
30	DB12877	Oxatomide	-31.41	-30.47	In vitro SARS-CoV-2 inhibition in Vero CCL-81 cells IC_{50} 25 µM and CC_{50} = 40µM.[16]
31	DB06555	Siramesine	-30.47	-31.53	…
32	DB14883	Lorecivivint	-30.41	-32.96	…
33	DB03966	Clorobiocin	-29.60	-31.92	…

No.	Drugbank ID	Name	ΔG_{MMPBSA} kcal/mol	ΔG_{thermo} kcal/mol	Experimental SARS-CoV-2 data
34	DB12306	Cipargamin	-29.47	-30.45	…
35	DB12566	Decernotinib	-29.31	-24.74	…
36	DB01260	Desonide	-29.18	-29.15	…
37	DB11925	Vistusertib	-28.97	-25.23	SARS-CoV-2 activity in Vero E6 cells, IC_{50} <25 nM.[17]
38	DB13791	Penfluridol	-28.75	-30.41	SARS-CoV-2 in vitro activity in Vero CCL-81 cells, EC_{50} 1.9 μM and CC_{50} = 3.3 μM.[16] In vitro activity in Vero E6 cells with IC_{50} = 2.4 μM and CC_{50} = 12.9 μM.[18]
39	DB15583	Fluazuron	-28.24	-31.42	…
40	DB07138	Neflamapimod	-28.03	-30.38	…
41	DB12703	Omipalisib	-27.98	-24.36	…
42	DB12264	Atevirdine	-27.89	-29.33	…
43	DB13434	Fenticonazole	-27.87	-23.12	…
44	DB13074	Macimorelin	-27.84	-22.34	…
45	DB00342	Terfenadine	-27.33	-28.88	SARS-CoV-2 IC_{50} = 3.0 μM in Vero cells.[19]
46	DB11616	Pirarubicin	-26.74	-26.33	IC_{50} = 4-7 μM in nsp15 FRET assay.[20]
47	DB01349	Tasosartan	-26.32	-26.44	…
48	DB15630	Glumetinib	-26.10	-24.85	…
49	DB00547	Desoximetasone	-26.09	-25.29	…
50	DB11977	Golvatinib	-25.65	-28.45	…
51	DB06589	Pazopanib	-25.58	-25.14	…
52	DB00568	Cinnarizine	-25.54	-25.10	SARS-CoV-2 activity in Vero CCL-81 cells, EC_{50}=40 μM, CC_{50} = 100μM.[16]
53	DB11830	Mocetinostat	-25.45	-27.99	…
54	DB00619	Imatinib	-25.36	-27.87	Inhibits SARS-CoV-2 with an IC_{50} = 130 nM in Vero cells.[21] EC_{50} = 2.5 μM, CC_{50} > 40μM in Vero E6 cells.[22] SARS-CoV-2 EC_{50}=4.9 μM, IC_{50}=37.3 μM by luciferase assay in lung organoids.[23]

No.	Drugbank ID	Name	ΔG_{MMPBSA} kcal/mol	ΔG_{thermo} kcal/mol	Experimental SARS-CoV-2 data
55	DB12427	Orvepitant	-25.36	-22.78	…
56	DB14895	Vibegron	-24.88	-20.32	…
57	DB13248	Phthalylsulfathiazole	-24.52	-26.74	…
58	DB13005	Rebastinib	-24.51	-22.52	…
59	DB06660	Saredutant	-24.48	-22.45	…
60	DB11986	Entrectinib	-24.23	-27.12	…
61	DB09003	Clocapramine	-24.22	-20.58	…
62	DB06446	Dotarizine	-24.13	-20.33	…
63	DB09143	Sonidegib	-24.11	-21.20	…
64	DB06077	Lumateperone	-23.87	-20.96	…
65	DB06638	Quarfloxin	-23.56	-27.18	…
66	DB13919	Candesartan	-23.21	-27.33	Inhibits human coronavirus (HCoV) strain OC43 propagated in LLC-MK2 cells, IC_{50} = 3.6μM, CC_{50} ~10μM.[3]
67	DB15391	Elenbecestat	-23.12	-25.63	…
68	DB08901	Ponatinib	-22.89	-27.03	Inhibition of SARS-CoV-2 in Huh7 cells engineered with the human ACE-2 receptor using immunofluorescence, EC_{50} = 1.1μM, CC_{50} = 8.7 μM.[24]
69	DB12412	Gemigliptin	-22.88	-20.79	…
70	DB12978	Pexidartinib	-22.87	-20.23	SARS-CoV-2 inhibition IC_{50} = 5.4 μM in Caco-2 cells.[25]
71	DB04908	Flibanserin	-22.47	-19.25	…
72	DB12562	Setipiprant	-22.32	-25.92	…
73	DB06212	Tolvaptan	-22.29	-25.47	…
74	DB13042	Fenoverine	-22.10	-23.44	…
75	DB06630	Anacetrapib	-21.98	-23.47	…
76	DB13814	Talampicillin	-21.64	-20.10	…
77	DB11904	Flumatinib	-21.25	-19.23	…
78	DB12492	Piritramide	-21.23	-18.53	…
79	DB13050	Tirilazad	-21.21	-25.31	…
80	DB11851	Bafetinib	-20.78	-22.35	SARS-CoV-2 inhibition in A549 cells over-expressing ACE2, EC_{50}

No.	Drugbank ID	Name	ΔG_{MMPBSA} kcal/mol	ΔG_{thermo} kcal/mol	Experimental SARS-CoV-2 data
					2.2 μM.[26]SARS-CoV-2 inhibition in SARS-CoV-2 titres in A549-ACE2 cells, IC_{50} = 790 nM.[27]
81	DB12121	Entospletinib	-20.54	-19.41	…
82	DB12414	Usistapide	-20.45	-19.74	…
83	DB14878	Liafensine	-20.23	-24.56	…
84	DB12154	Itacitinib	-19.74	-22.45	…
85	DB15444	Elexacaftor	-19.32	-23.22	…
86	DB15006	Flufenoxuron	-19.30	-23.12	…
87	DB12465	Ketanserin	-18.45	-20.98	…

References Listed in Supplementary Table S1

1. Touret F, Gilles M, Barral K, Nougairède A, Decroly E, de Lamballerie X, et al. In vitro screening of a FDA approved chemical library reveals potential inhibitors of SARS-CoV-2 replication. *bioRxiv.* 2020:2020.2004.2003.023846. doi:10.1101/2020.04.03.023846
2. Bellavite P, Donzelli A. Hesperidin and SARS-CoV-2: New light on the healthy function of citrus fruits. *Antioxidants.* 2020;9(8):742.
3. Xiao X, Wang C, Chang D, Wang Y, Dong X, Jiao T, et al. Identification of potent and safe antiviral therapeutic candidates against SARS-CoV-2. *bioRxiv.* 2020:2020.2007.2006.188953. doi:10.1101/2020.07.06.188953
4. Xie X, Muruato AE, Zhang X, Lokugamage KG, Fontes-Garfias CR, Zou J, et al. A nanoluciferase SARS-CoV-2 for rapid neutralization testing and screening of anti-infective drugs for COVID-19. *bioRxiv.* 2020:2020.06.22.165712. doi:10.1101/2020.06.22.165712.
5. Kuo CJ, Chao TL, Kao HC, Tsai YM, Liu YK, Wang LH, et al. Kinetic characterization and inhibitor screening for the proteases leading to identification of drugs against SARS-CoV-2. *Antimicrob Agents Chemother.* 2021. doi:10.1128/AAC.02577-20
6. Pickard A, Calverley BC, Chang J, Garva R, Lu Y, Kadler KE. Discovery of re-purposed drugs that slow SARS-CoV-2 replication in human cells. *bioRxiv.* 2021.
7. Yamamoto N, Matsuyama S, Hoshino T, Yamamoto N. Nelfinavir inhibits replication of severe acute respiratory syndrome coronavirus 2 in vitro. *bioRxiv.* 2020:2020.2004.2006.026476. doi:10.1101/2020.04.06.026476
8. Vatansever EC, Yang KS, Drelich AK, Kratch KC, Cho C-C, Kempaiah KR, et al. Bepridil is potent against SARS-CoV-2 in vitro. *Proceedings of the National Academy of Sciences.* 2021;118(10):e2012201118. doi:10.1073/pnas.2012201118
9. Rizzuti B, Grande F, Conforti F, Jimenez-Alesanco A, Ceballos-Laita L, Ortega-Alarcon D, et al. Rutin is a low micromolar inhibitor of SARS-CoV-2 main protease 3CLpro: Implications for drug design of quercetin analogs. *Biomedicines.* 2021;9(4):375. https://www.mdpi.com/2227-9059/9/4/375.
10. Weston S, Coleman CM, Haupt R, Logue J, Matthews K, Frieman MB. Broad anti-coronaviral activity of FDA approved drugs against SARS-CoV-2 in vitro and SARS-CoV in vivo. *bioRxiv.* 2020:2020.2003.2025.008482. doi:10.1101/2020.03.25.008482
11. Dyall J, Coleman CM, Hart BJ, Venkataraman T, Holbrook MR, Kindrachuk J, et al. Repurposing of clinically developed drugs for treatment of middle east respiratory syndrome coronavirus infection. *Antimicrobial Agents and Chemotherapy.* 2014;58(8):4885-4893. doi:10.1128/aac.03036-14
12. Raymonda MH, Ciesla JH, Monaghan M, Leach J, Asantewaa G, Smorodintsev-Schiller LA, et al. Pharmacologic profiling reveals lapatinib as a novel antiviral against SARS-CoV-2 in vitro. *bioRxiv.* 2020:2020.2011.2025.398859. doi:10.1101/2020.11.25.398859

13. Riva L, Yuan S, Yin X, Martin-Sancho L, Matsunaga N, Burgstaller S, et al. A large-scale drug repositioning survey for SARS-CoV-2 antivirals. *BioRxiv.* 2020.
14. Malik S, Gupta A, Zhong X, Rasmussen TP, Manautou JE, Bahal R. Emerging therapeutic modalities against COVID-19. *Pharmaceuticals (Basel).* 2020;13(8):188. doi:10.3390/ph13080188
15. Ku KB, Shin HJ, Kim HS, Kim B-T, Kim S-J, Kim C. Repurposing screens of FDA-approved drugs identify 29 inhibitors of SARS-CoV-2. *J Microbiol Biotechnol.* 2020;30(12):1843-1853. doi:10.4014/jmb.2009.09009
16. Shin JS, Jung E, Jang Y, Han SB, Kim M. Identification of diphenoxylate as an antiviral agent against severe acute respiratory syndrome coronavirus 2. *ChemRxiv.* 2020(June):chemrxiv.12496547.v1 doi:doi:10.26434/chemrxiv.12496547.v1
17. Garcia Jr G, Sharma A, Ramaiah A, Sen C, Kohn DB, Gomperts BN, et al. Antiviral drug screen of kinase inhibitors identifies cellular signaling pathways critical for SARS-CoV-2 replication. *Cell Reports.* 2020:in press. doi:10.2139/ssrn.3682004
18. Jan J-T, Cheng T-JR, Juang Y-P, Ma H-H, Wu Y-T, Yang W-B, et al. Identification of existing pharmaceuticals and herbal medicines as inhibitors of SARS-CoV-2 infection. *Proceedings of the National Academy of Sciences.* 2021;118(5):e2021579118. doi:10.1073/pnas.2021579118
19. Killick R, Ballard C, Doherty P, Williams G. Using the SARS infection transcriptional signature to identify potential treatments for covid-19. *ChemRxiv.* 2020(May):chemrxiv.12327554.v1 doi:10.26434/chemrxiv.12327554.v1
20. Choi R, Zhou M, Shek R, Wilson JW, Tillery L, Craig JK, et al. High-throughput screening of the reframe, pandemic box, and COVID box drug repurposing libraries against SARS-cov2 nsp15 endoribonuclease to identify small-molecule inhibitors of viral activity. *bioRxiv.* 2021:2021.2001.2021.427657. doi:10.1101/2021.01.21.427657
21. Mulgaonkar N, Wang H, Mallawarachchi S, Fernando S, Martina B, Ruzek D. Bcr-abl tyrosine kinase inhibitor imatinib as a potential drug for COVID-19. *bioRxiv.* 2020:2020.2006.2018.158196. doi:10.1101/2020.06.18.158196
22. Touret F, Driouich J-S, Cochin M, Rémi Petit P, Gilles M, Barthélémy K, et al. Preclinical evaluation of imatinib does not support its use as an antiviral drug against SARS-CoV-2. *bioRxiv.* 2020:2020.2011.2017.386904. doi:10.1101/2020.11.17.386904
23. Han Y, Yang L, Duan X, Duan F, Nilsson-Payant BE, Yaron TM, et al. Identification of candidate COVID-19 therapeutics using hpsc-derived lung organoids. *bioRxiv.* 2020:2020.2005.2005.079095. doi:10.1101/2020.05.05.079095
24. Milani M, Donalisio M, Bonotto RM, Schneider E, Arduino I, Boni F, et al. Combined in silico and in vitro approaches identified the antipsychotic drug lurasidone and the antiviral drug elbasvir as SARS-cov2 and hcov-oc43 inhibitors. *Antiviral Research.* 2021;189:105055. doi:https://doi.org/10.1016/j.antiviral.2021.105055
25. Ellinger B, Bojkova D, Zaliani A, Cinatl J, Claussen C, Westhaus S, et al. Identification of inhibitors of SARS-CoV-2 in-vitro cellular toxicity in human (caco-2) cells using a large scale drug repurposing collection. *Research Square.* 2021:s-23951/v1. doi:10.21203/rs.3.rs-23951/v1. Accessed 2021/04/14.

26. Drayman N, Jones KA, Azizi S-A, Froggatt HM, Tan K, Maltseva NI, et al. Drug repurposing screen identifies masitinib as a 3CLpro inhibitor that blocks replication of SARS-CoV-2 in vitro. *bioRxiv.* 2020:2020.2008.2031.274639. doi:10.1101/2020.08.31.274639
27. Meyer B, Chiaravalli J, Gellenoncourt S, Brownridge P, Bryne DP, Daly LA, et al. Characterisation of protease activity during SARS-CoV-2 infection identifies novel viral cleavage sites and cellular targets for drug repurposing. *bioRxiv.* 2021:2020.2009.2016.297945. doi:10.1101/2020.09.16.297945

Table S2. Description of top 20 drugs predicted to bind and inhibit SARS-CoV-2 helicase.

Drug	Structure	Description	Binding Energy (kcal/mol)
Dolutegravir (antiretrovira l)	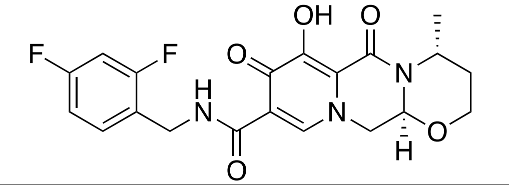	An integrase inhibitor used to treat HIV/AIDS	-42.2
Hesperidin (citrus flavanone glycoside)	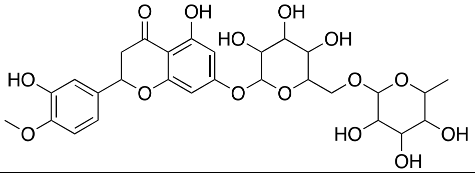	Hesperidin is a bioflavonoid antioxidant obtained from citrus plants	-41.2
Cabotegravir (antiviral integrase inhibitor)	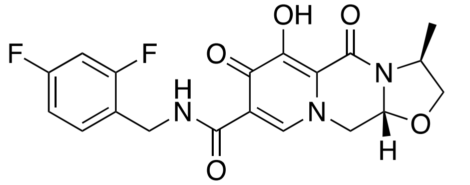	An HIV integrase inhibitor with a carbamoyl pyridone structure similar to that of dolutegravir	-40.8
Conivaptan (vasopressin inhibitor)	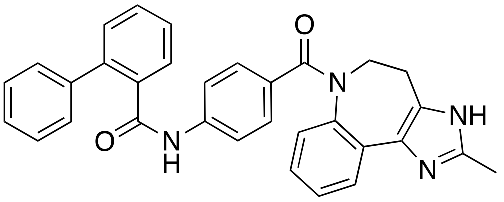	Dual inhibitor of the vasopressin receptors, V1a and V2	-40.2
Aprepitant (NK1 antagonist)	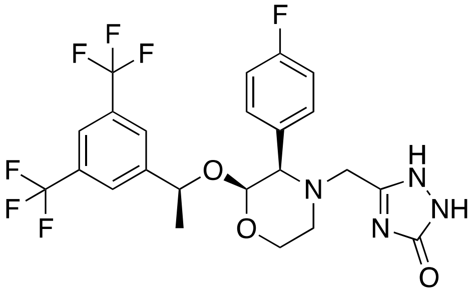	Selective antagonist of the neurokinin-1 receptor used to prevent chemotherapy-induced nausea and vomiting and to prevent postoperative nausea and vomiting.	-40.1
Bictegravir (antiviral integrase inhibitor)	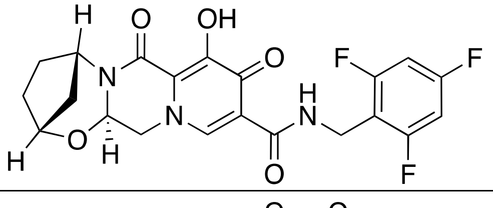	HIV integrase inhibitor structurally derived from dolutegravir	-39.8
Manidipine (Ca channel blocker, anti-hypertensive)	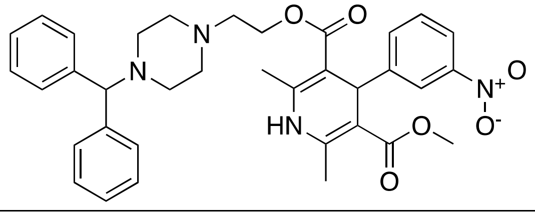	Dihydropyridine type calcium channel blocker used clinically as an antihypertensive	-39.8
Tipranavir (antiviral protease inhibitor)	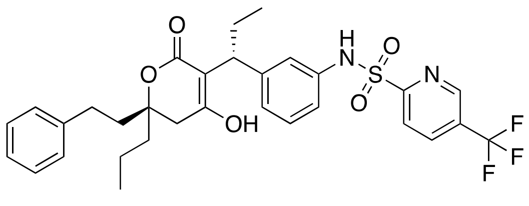	Nonpeptidic protease inhibitor administered with ritonavir in combination therapy to treat HIV infection	-39.7

Aminoquinuride (trypanocidal agent)	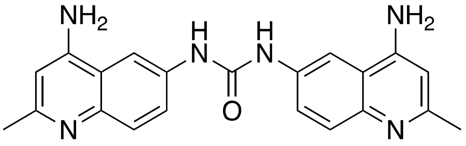	Belongs to the class of organic compounds known as 4-aminoquinolines that inhibit cellular glycosaminoglycan expression thereby inhibiting microbial attachment and host cell entry of trypanosomes	-39.6
Antrafenine (analgesic anti-inflammatory)	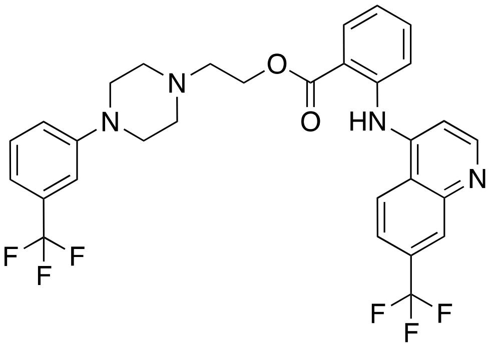	Phenylpiperazine derivative drug that acts as an analgesic and anti-inflammatory	-39.1
RSV-604 (antiviral)	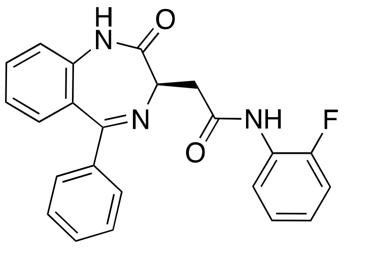	A benzodiazepine with submicromolar anti-RSV activity that act via interaction with the viral nucleocapsid protein	-38.8
Epirubicin (anticancer intercalator)	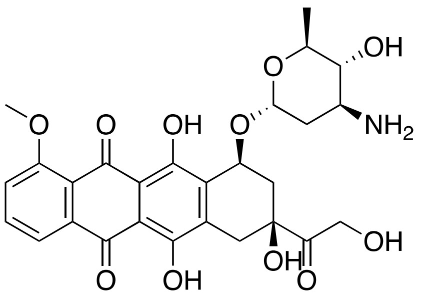	Anthracycline drug used for chemotherapy which acts by intercalating DNA resulting in complex formation which inhibits DNA and RNA synthesis and triggers DNA cleavage by topoisomerase II resulting in cell death.	-38.7
Pimozide (antipsychotic)	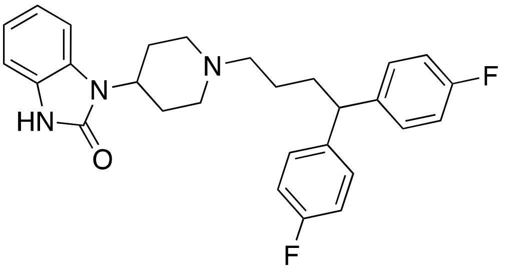	Antipsychotic drug of the diphenylbutylpiperidine class that selectively inhibits type 2 dopaminergic receptors and also antagonizes alpha-adrenergic and 5-HT2 receptors	-38.4

Rutin (flavonol glycoside)	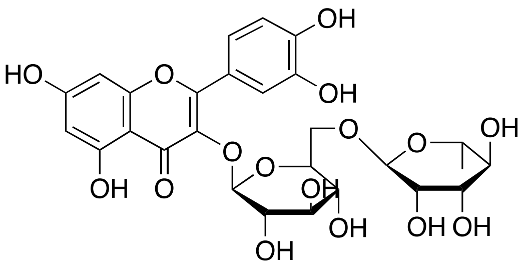	Quercetin-3-O-rutinoside is a citrus flavonoid glycoside that combines the flavonol quercetin and the disaccharide rutinose.It has antioxidant properties and is used in natural medicines	-38.3
Dicoumarol (anticoagulant)	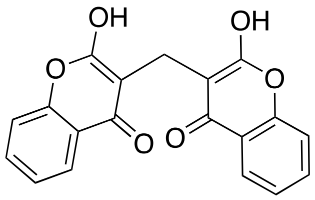	Naturally occurring anticoagulant drug that depletes liver stores of vitamin K and inhibits reductases	-37.6
Fluspirilene (antipsychotic)	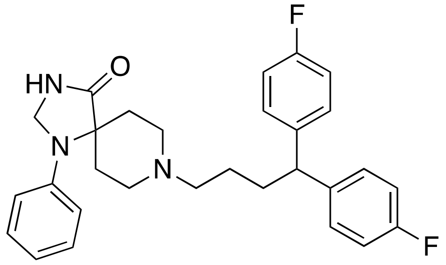	Diphenylbutylpiperidine antipsychotic drug used for the treatment of schizophrenia that also targets glioma stem cells in glioblastoma through inactivating signal transducer and activator of transcription 3 (STAT3)	-36.8
Doramapimod (p38 MAP kinase inhibitor)	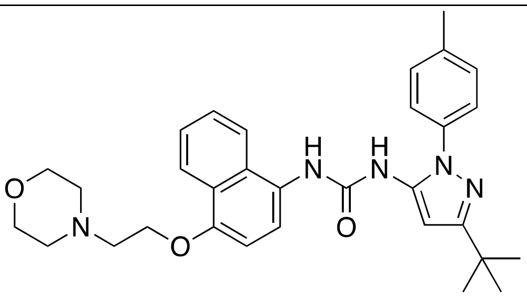	Pyrazoles that is used for treatment of rheumatoid arthritis, Crohn's disease and psoriasis due to its ability to inhibit p38 mitogen-activated protein kinase	-36.2
Fexofenadine (antihistamine)	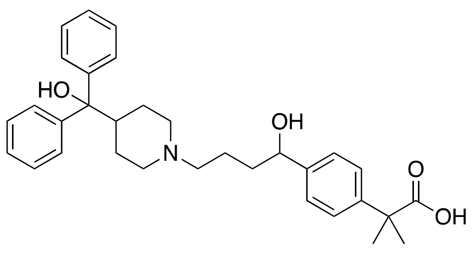	Antihistamine with selective peripheral H1-receptor antagonist activity used in the treatment of allergy symptoms, such as hay fever and urticaria	-35.5
Astemizole (antihistamine)	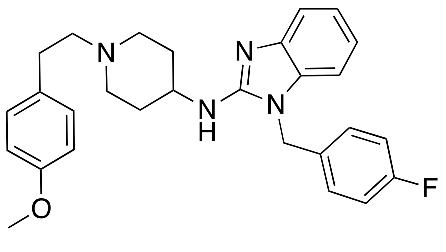	Antihistamine with H1-receptor antagonist activity that is also a hERG potassium channel blocker	-35.4

Sertindole (antipsychotic)	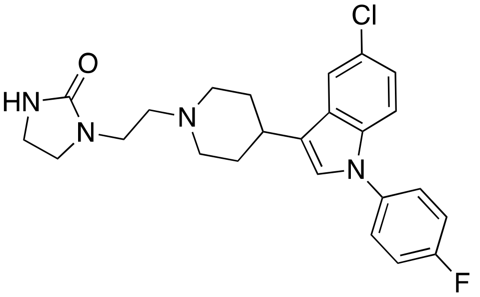	Atypical antipsychotic with activity at dopamine and serotonin receptors in the brain	-34.6

Supplementary Table S3. Ligplots showing how top 20 hits in alphabetical order interact with residues in SARS-CoV-2 helicase.

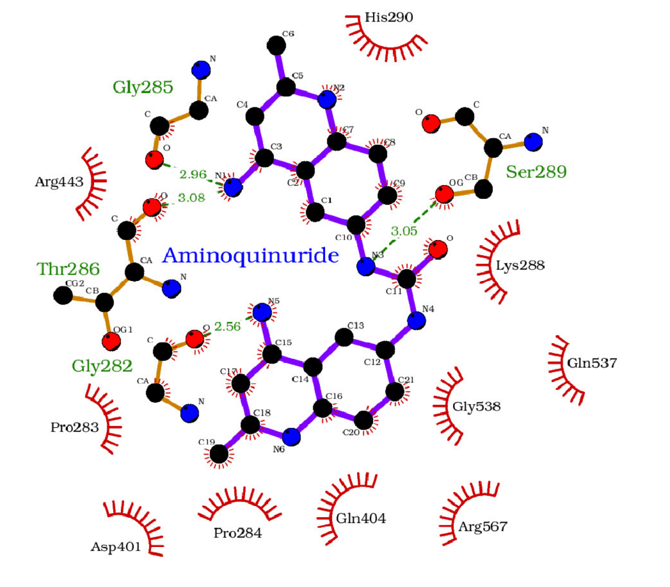

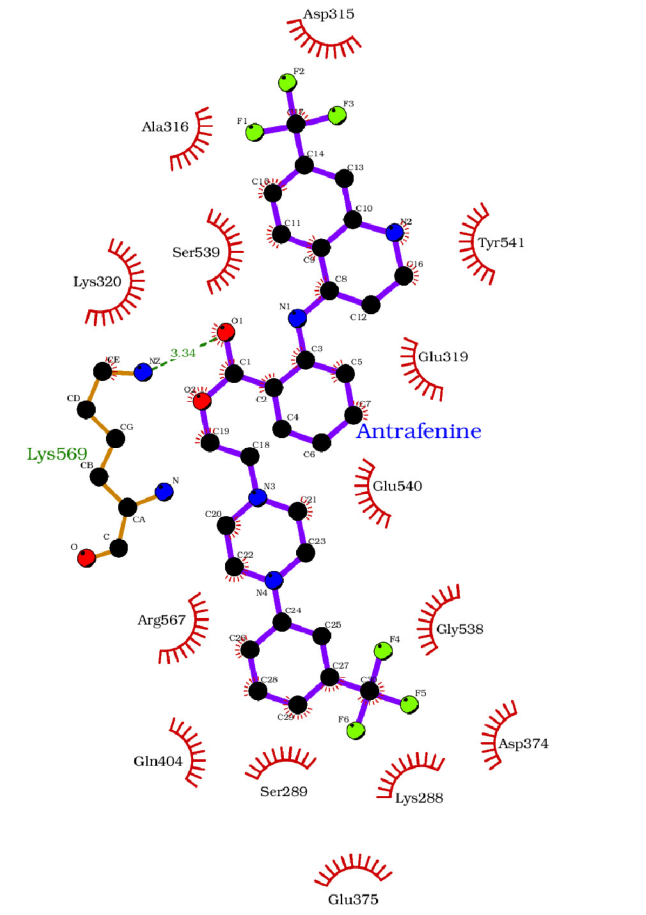

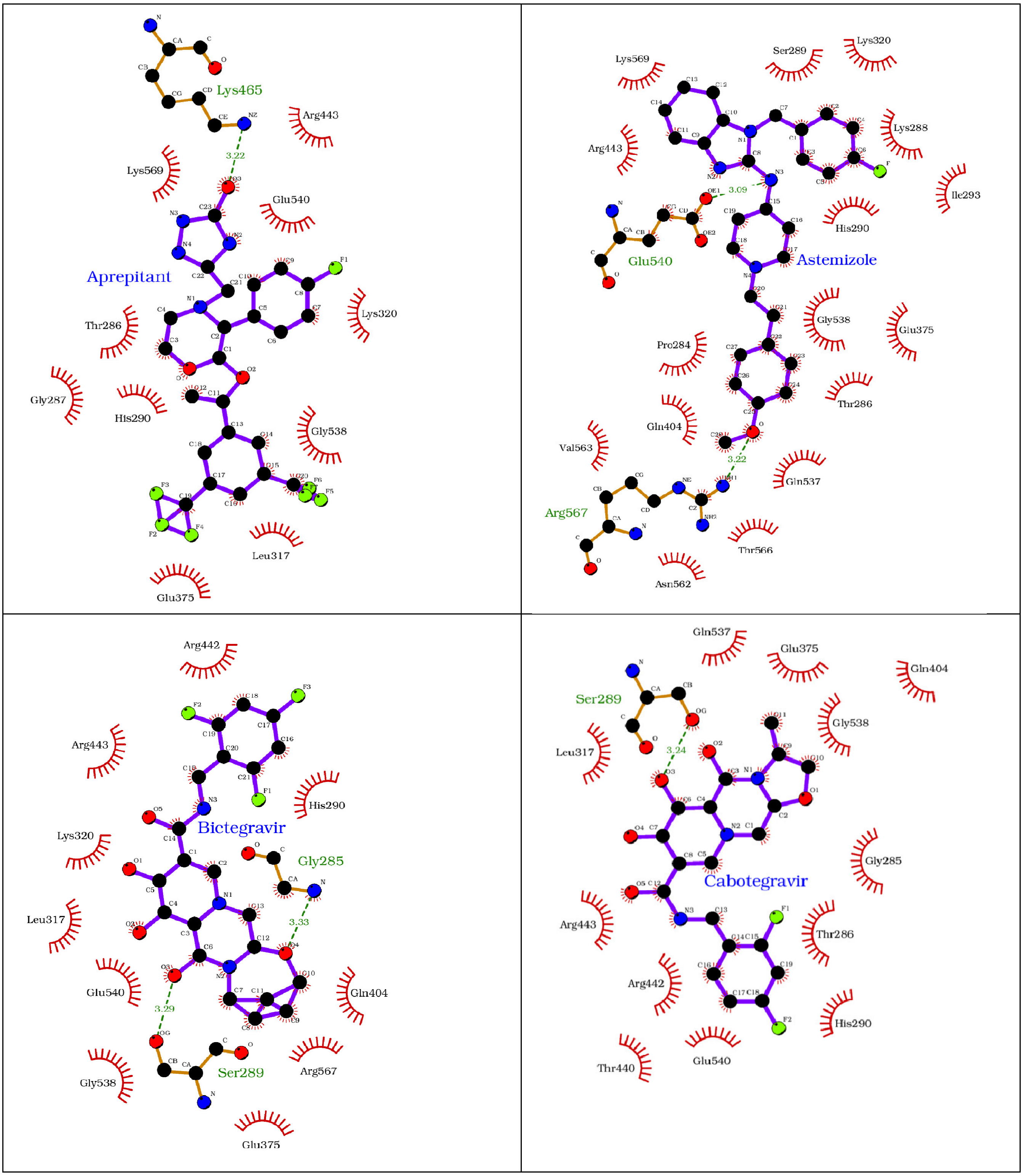
Lys465
Arg443
Lys569
3.22
Glu540
Aprepitant
Thr286
Lys320
Gly287
His290
Gly538
Leu317
Glu375
Lys569
Ser289
Lys320
Arg443
Lys288
Ile293
3.09
His290
Glu540
Astemizole
Gly538
Glu375
Pro284
Thr286
Gln404
Val563
3.22
Gln537
Arg567
Thr566
Asn562
Arg442
Arg443
His290
Lys320
Bictegravir
Gly285
Leu317
3.33
Glu540
Gln404
3.29
Ser289
Arg567
Gly538
Glu375
Gln537
Glu375
Gln404
Ser289
Gly538
Leu317
3.24
Gly285
Cabotegravir
Arg443
Thr286
Arg442
His290
Thr440
Glu540

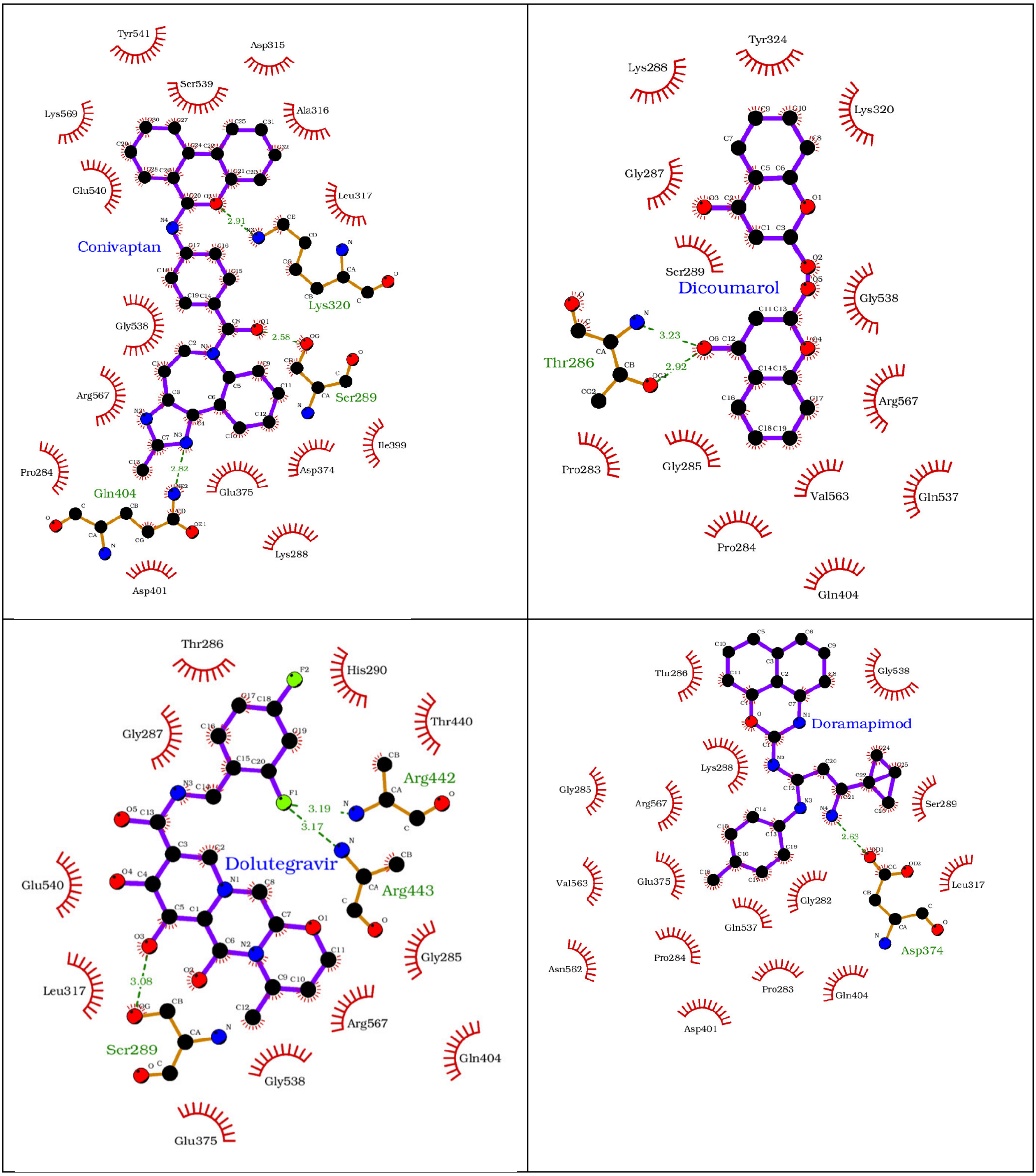
Tyr541
Asp315
Ser539
Lys569
Ala316
Glu540
Leu317
2.91
Conivaptan
Lys320
Gly538
2.58
Ser289
Arg567
Ile399
Pro284
Asp374
2.82
Gln404
Glu375
Lys288
Asp401
Tyr324
Lys288
Lys320
Gly287
Ser289
Dicoumarol
Gly538
3.23
Thr286
2.92
Arg567
Pro283
Gly285
Val563
Gln537
Pro284
Gln404
Thr286
His290
Thr440
Gly287
Arg442
3.19
3.17
Dolutegravir
Arg443
Glu540
Gly285
3.08
Leu317
Arg567
Ser289
Gln404
Gly538
Glu375
Thr286
Gly538
Doramapimod
Lys288
Gly285
Arg567
Ser289
2.63
Val563
Glu375
Leu317
Gly282
Gln537
Asp374
Asn562
Pro284
Pro283
Gln404
Asp401

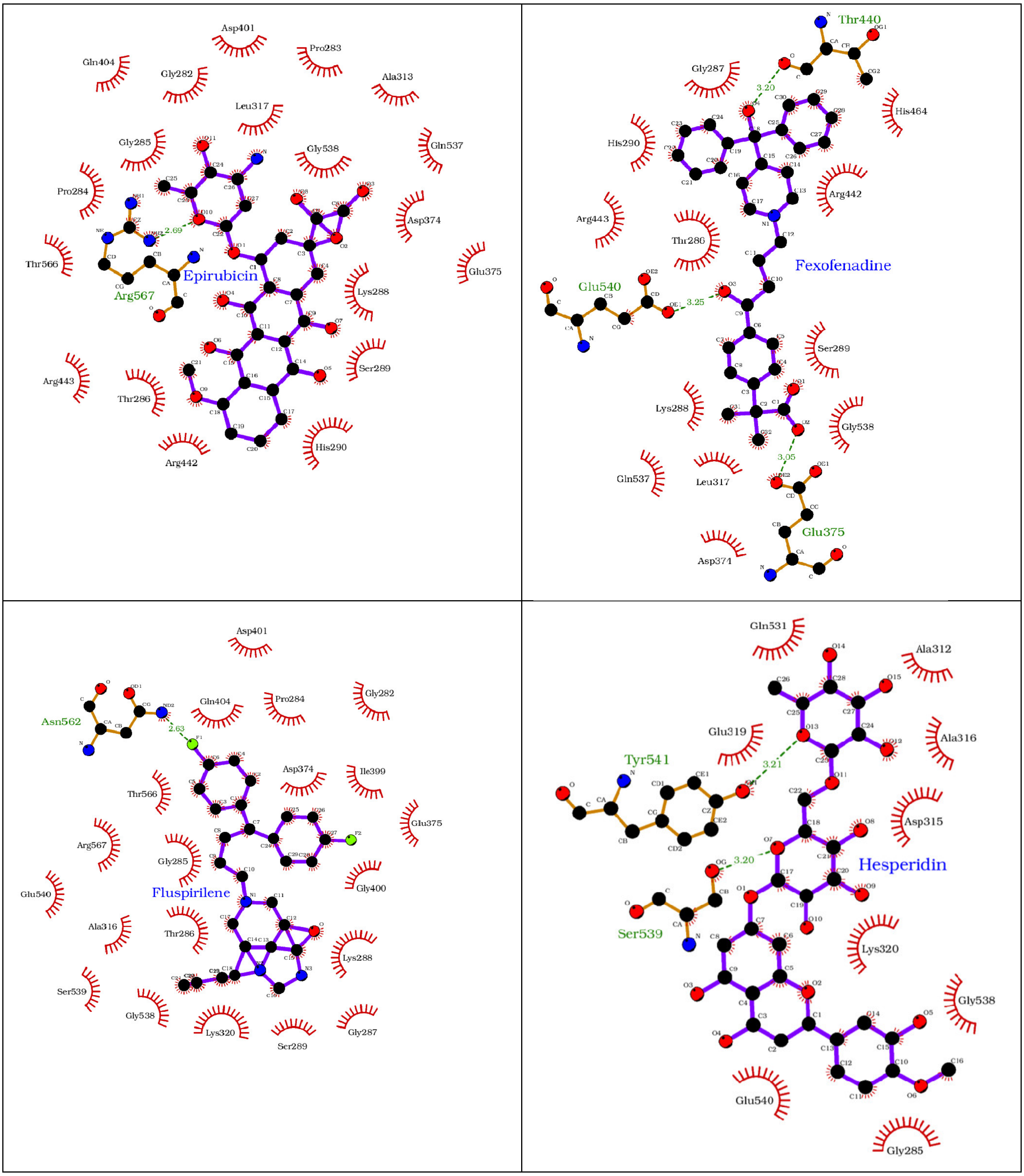
Asp401
Pro283
Gln404
Gly282
Ala313
Leu317
Gly285
Gly538
Gln537
Pro284
Asp374
2.69
Thr566
Glu375
Epirubicin
Arg567
Lys288
Arg443
Ser289
Thr286
His290
Arg442
Thr440
Gly287
3.20
His464
His290
Arg442
Arg443
Thr286
Fexofenadine
Glu540
3.25
Ser289
Lys288
Gly538
3.05
Gln537
Leu317
Glu375
Asp374
Asp401
Gln404
Pro284
Gly282
Asn562
2.63
Asp374
Ile399
Thr566
Glu375
Arg567
Gly285
Gly400
Fluspirilene
Glu540
Ala316
Thr286
Lys288
Ser539
Gly538
Lys320
Gly287
Ser289
Gln531
Ala312
Glu319
Ala316
Tyr541
3.21
Asp315
3.20
Hesperidin
Ser539
Lys320
Gly538
Glu540
Gly285

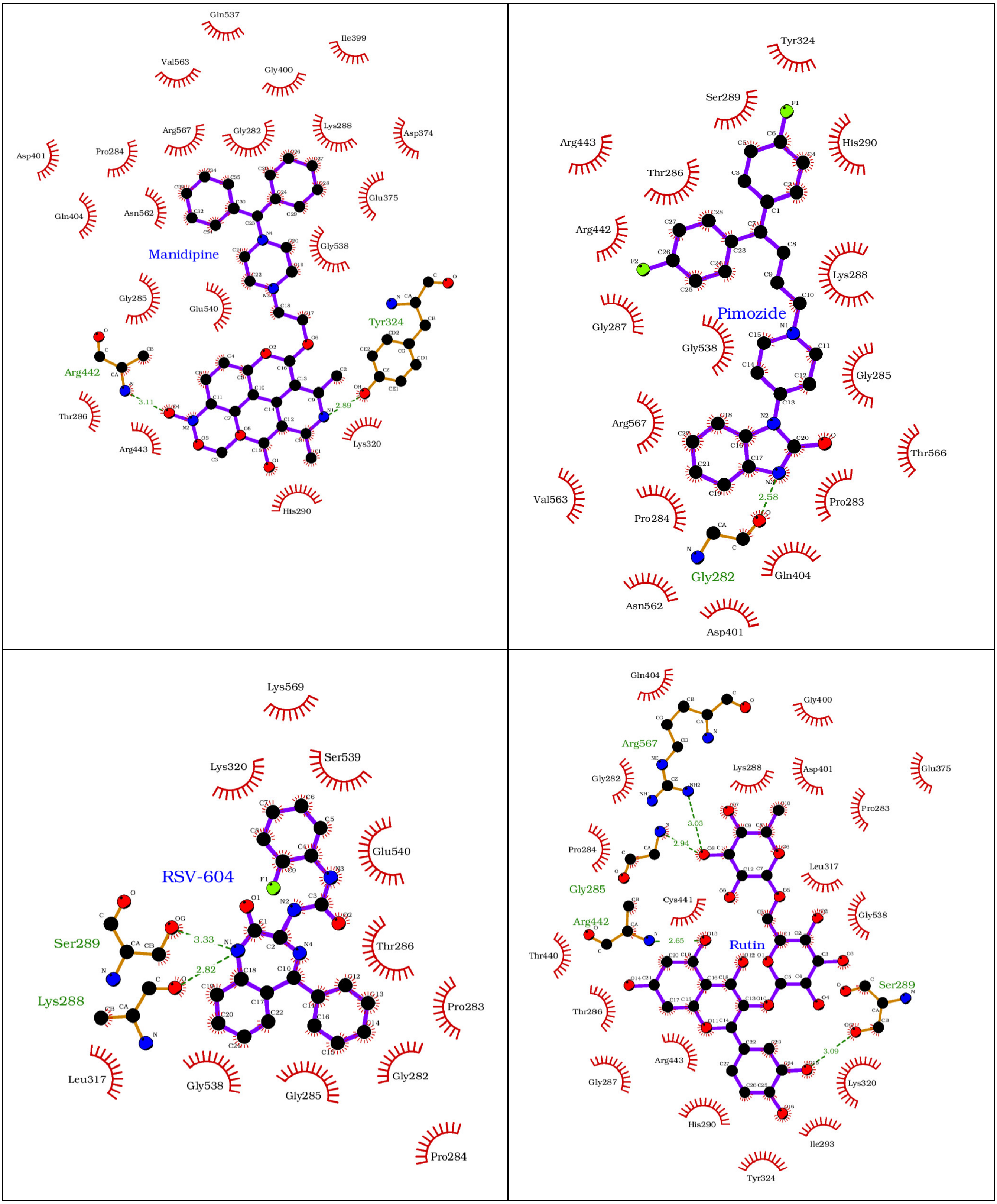
Gln537
Ile399
Val563
Gly400
Arg567
Gly282
Lys288
Asp374
Asp401
Pro284
Gln404
Asn562
Glu375
Manidipine
Gly538
Gly285
Glu540
Tyr324
Arg442
3.11
2.89
Thr286
Arg443
Lys320
His290
Tyr324
Ser289
His290
Arg443
Thr286
Arg442
Lys288
Gly287
Pimozide
Gly538
Gly285
Arg567
Thr566
Val563
Pro284
2.58
Pro283
Gly282
Gln404
Asn562
Asp401
Lys569
Ser539
Lys320
Glu540
RSV-604
Ser289
3.33
Thr286
2.82
Lys288
Pro283
Leu317
Gly538
Gly285
Gly282
Pro284
Gln404
Gly400
Arg567
Lys288
Asp401
Glu375
Gly282
Pro283
3.03
2.94
Pro284
Leu317
Gly285
Cys441
Gly538
Arg442
2.65
Rutin
Thr440
Ser289
Thr286
3.09
Arg443
Lys320
Gly287
His290
Ile293
Tyr324

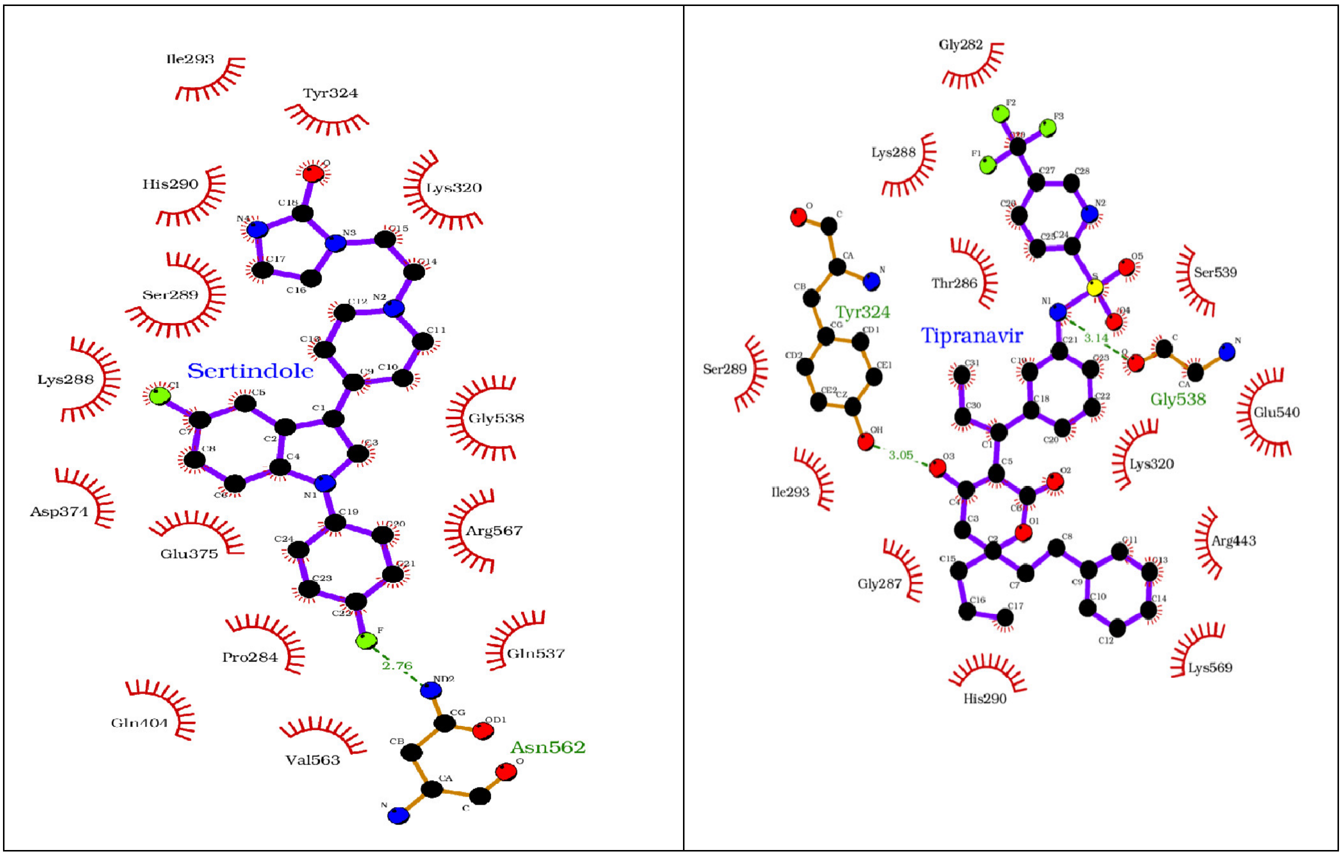
Sertindole
Ile293
Tyr324
His290
Lys320
Ser289
Lys288
Gly538
Asp374
Glu375
Arg567
Pro284
Gln537
Gln404
Val563
Asn562
2.76
Tipranavir
Gly282
Lys288
Tyr324
Thr286
Ser539
Ser289
Gly538
Glu540
Ile293
Lys320
Arg443
Gly287
His290
Lys569
3.14
3.05

Supplementary Table S4. Scripts Used in Modelling.

1)Conf.txt

```
receptor = 6Y2F.pdbqt
center_x=  9.245
center_y=  -0.788
center_z = 18.371
size_x = 50
size_y = 50
size_z = 50
num_modes = 10
exhaustiveness = 50
```

2)vina_screen.sh

```
#! /bin/bash
for f in CHEMBL*.pdbqt; do
   b=`basename $f .pdbqt`
   echo Processing ligand $b
mkdir -p $b
   vina --config conf.txt --cpu 50 --ligand $f --out $[b]/out.pdbqt --log $[b]/log.txt
done
```

3)Script1.py

```
#! /usr/bin/env python
import sys
import glob
def doit(n):
file_names = glob.glob('*/*.pdbqt')
   everything = []
   failures = []
   print 'Found', len(file_names), 'pdbqt files'
   for file_name in file_names:
      file = open(file_name)
      lines = file.readlines()
file.close()
      try:
         line = lines[1]
         result = float(line.split(':')[1].split()[0])
everything.append([result, file_name])
      except:
failures.append(file_name)
everything.sort(lambda x,y: cmp(x[0], y[0]))
   part = everything[:n]
   for p in part:
```

```
        print p[1],
    print
    if len(failures) > 0:
        print 'WARNING:', len(failures), 'pdbqt files could not be processed'
if __name__ == '__main__':
doit(int(sys.argv[1]))
```
